# Moderation with a latent class variable: A tutorial and example

**DOI:** 10.3758/s13428-025-02886-x

**Published:** 2026-04-10

**Authors:** Dina Ali Naji Arch, Karen Nylund-Gibson, Marsha Ing

**Affiliations:** 1 Gevirtz School of Education, University of California, Santa Barbara, CA 93106 USA; 2 University of California, 900 University Ave, Riverside, CA 92521 USA

**Keywords:** Latent class moderation, Latent class analysis, Tutorial, Moderation

## Abstract

Moderation analyses allow a more nuanced understanding of the relationship between a predictor and an outcome. A limitation of traditional moderation analysis arises when addressing the hypothesis when using a moderator that does not account for unobserved heterogeneity in the population. This limitation can be addressed by extending moderation analyses to include a latent class analysis with auxiliary variables (as predictors and/or outcomes) where the moderator is the latent class variable. This tutorial specifies this extended moderation model with a latent class variable using the three-step manual approach (Asparouhov & Muthén, *Structural Equation Modeling: A Multidisciplinary Journal*, *21*, 329-341, 2014). Data from the Longitudinal Survey of American Life illustrates this approach within the context of science attitudes. Specifically, a latent class variable (science attitudes) is hypothesized to moderate the relationship between a predictor (science achievement) and an outcome (interest in science issues), while controlling for demographic variables.

**Supplementary Information:**

The online version contains supplementary material available at 10.3758/s13428-025-02886-x.

## Introduction

Statistical moderation provides a nuanced framework for understanding the relationship between predictors and outcomes, as well as how these relationships may vary across different levels of a moderating variable. In the social sciences, moderation is widely recognized as an important method for exploring the conditional relationships between predictors and the outcomes of interest. Substantial methodological efforts have been dedicated to expanding traditional moderation techniques, encompassing various models, from standard multiple regression involving interactions between continuous or categorical variables, to more intricate ones incorporating three-way and non-linear interactions. These models can also integrate categorical and nominal moderators and incorporate mediational contexts (such as moderated mediation). However, a challenge in these approaches emerges when traditional moderation analyses address hypotheses involving unobserved heterogeneity in a population with a measured or observed moderator. In these cases, relying on a single observed moderator may mask meaningful subgroup differences and can lead to incomplete conclusions about moderation effects.

Our focus is on methodological advancements designed to address increasingly complex hypotheses, encompassing both observed (measured) and unobserved (latent) variables. Researchers use mixture modeling approaches, such as latent class analysis (LCA), to model the unobserved heterogeneity in a population and can investigate how external factors influence or predict latent class membership by relating covariates to the latent class model (Vermunt, 2010; Asparouhov & Muthén, 2014). In scenarios where interactions with unobserved heterogeneity are of interest, researchers enhance the moderation model by introducing a latent class variable as the moderator. This tutorial aims to illustrate the benefits of such an extension, specifically focusing on mixture models incorporating a categorical latent variable, or LCA variable.

LCA is part of a more extensive mixture modeling family of models and has become a widely utilized statistical method in social science research used to identify unobserved subgroups within a population (Masyn, 2013; Nylund-Gibson & Choi, 2018). LCA uses a categorical or binary set of indicators to estimate the model and identify subgroups or “classes.” One of the unique contributions of LCA is that it identifies heterogeneity in a population—that is, the model assumes that there are unobserved subpopulations in a given population that can be identified using a set of observed variables. Identifying the unobserved subpopulations provides a more nuanced understanding of the population and its relations. For a comprehensive overview of LCA, refer to Nylund-Gibson and Choi (2018), Masyn (2013), and Lanza and Cooper (2016).

Recent developments in research applications have included the latent class variable as a moderator. In these contexts, the modeling includes both a predictor and outcome, where moderation occurs by estimating regression parameters for each latent class (McLarnon & O’Neill, 2018; Asparouhov & Muthén, 2014; Nylund-Gibson et al., 2022), referred to as *latent class moderation*. Latent class moderation is helpful to researchers who hypothesize that the moderation relationship may not be linear, that is, when the moderation effect varies across groups of individuals in the population. This is a departure from the more conventional perspective on moderation, where the effect of the moderator either increases or decreases for each unit increase of the moderator. When using a latent class moderator, more information is learned about the linear relationships between a predictor and an outcome across subsets of individuals. This focus on individual variation (person-centered) in moderation is unique, whereas traditional moderation in the regression context is not person-centered.

Several recent applications of moderation with a latent class variable have been reported. Felix and colleagues (2019) used a latent class variable to investigate how different flood exposure experiences changed the nature of the relationship between life stress and social-emotional outcomes in youth. After identifying the latent classes of disaster exposure (high, moderate, community, and low exposure), they examined differences in the relations between life stressors (predictor) and social-emotional health (distal outcome) across different levels of disaster exposure (latent classes). By studying the difference in these relations across the different latent classes, they identified meaningful differences—specifically, life stressors have a qualitatively different impact on the well-being of the youth who had high disaster exposure. In contrast, the other exposure groups had similar relations. This example of a person-centered approach to moderation allows the regression relations to vary across the latent disaster exposure groups.

### Latent class moderation: Moderation with a latent class variable

This paper focuses on moderation by a latent class variable, in which the slope and intercept of a regression model vary across pre-defined latent classes. Consider a scenario where a continuous outcome $y$ is regressed on the covariate $x$ for individual $i$, with relationships allowed to vary across $K$ latent classes (denoted by categorical variable *C*, where $c = 1, 2, \dots , K$). The latent class moderation model can be expressed as,

1 $${y}_{i}|{C}_{i=c}={\beta }_{0c}+{\beta }_{1c}{x}_{i}+{r}_{i},$$

where the residual ${r}_{i}\sim N(0,{\theta }_{c})$, with the residual variance ${\theta }_{c}$ is typically held to be class invariant. Additionally, the intercept ${\beta }_{0}$ and slope ${\beta }_{1}$ are allowed to vary across classes. Moderation occurs in this context when the slope parameter significantly differs across classes.

### Comparison of regression mixture and latent class moderation models

The specification of the *latent class moderation* model involves estimating the regression relationship for each predefined latent class, allowing both the intercept and slope of a linear regression to vary across classes (Asparouhov & Muthén, 2014). In this framework, class membership is defined a priori—based on theory, prior analyses (such as latent class analysis), or other external information. Moderation is tested by examining whether regression parameters (e.g., slopes and intercepts) significantly differ across these known classes.

**Fig. 1:**
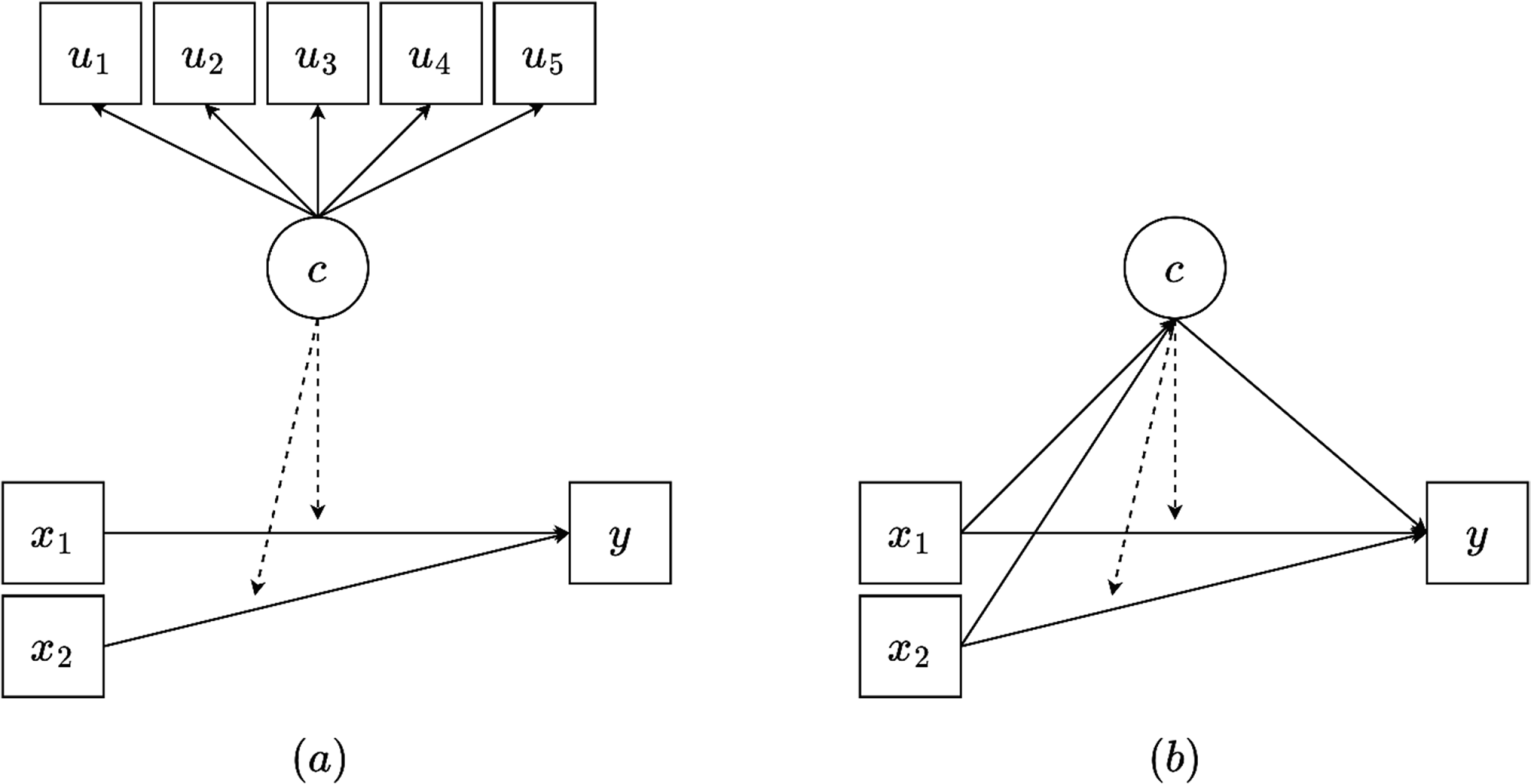
Path diagram (**a**) of the latent class moderation model, and path diagram (**b**) of the regression mixture model. The dotted arrow from the latent class variable *C* to the regression of outcome *y* on predictor *x* indicates that the slope and intercept vary across the latent classes

This analysis differs from a similar model, *regression mixture* models (e.g., Ding, 2006; Van Horn et al., 2015), in which classes are not predetermined but instead are formed based on the variability in the regression parameters themselves (e.g., intercept and slope heterogeneity). In *regression mixture models*, class membership is defined in part by qualitative differences in the relationship between predictor(s) and outcome(s), that is, by differences in the intercepts and/or slopes across latent classes (Kim et al., 2016; Van Horn et al., 2015). Table 1 and Fig. 1 highlight the differences between latent class moderation and the regression mixture model.

**Table 1:** Comparisons of latent class moderation and the regression mixture model

Feature	Latent class moderation	Regression mixture
When to use	Comparing regression across known groups	Exploring unknown groups with different regression parameters
Regression equation	Same equation (1); parameters vary by known class; class-specific regression coefficients are estimated separately	Same equation (1); variability in the regression coefficients defines the latent classes
How classes are defined	Based on a set of observed indicators (i.e., not included in the regression)	Based on differences in regression parameters (i.e., slopes, intercepts)
Class membership	Established before specifying the latent class moderation model (e.g., unconditional enumeration or theory)	Established using regression parameters (e.g., regression heterogeneity defines classes)

In contrast, *latent class moderation* models, as implemented in this paper, estimate separate regression models conditional on an existing latent class variable, defined by a set of observed indicators. These observed latent class indicators are not included in the regression model approach. That is, observed indicators define the latent class variable, whereas the heterogeneity in regression parameters defines the latent class variable in regression mixture. In short, *regression mixture* models are used to identify latent groups based on parameter differences, while *latent class moderation* models apply regression separately within pre-established classes. The choice between a *regression mixture* model and a *latent class moderation* model (which is the focus of the current paper) depends on the nature of the research question and the assumptions about heterogeneity in the data. A *regression mixture* model is more exploratory and is appropriate when the goal is to uncover *unobserved subpopulations* that differ in how predictors relate to outcome, while *latent class moderation* models are more confirmatory in nature and appropriate when the goal is to compare regression relationships across classes.

### Estimating a latent class moderation model

To estimate the latent class moderation model, the first step involves enumerating the latent classes, following the modeling conventions recommended in the mixture modeling literature (Nylund-Gibson & Masyn, 2016; Masyn, 2013). After class enumeration, the three-step maximum likelihood (ML) estimation procedure is employed, as outlined by Vermunt (2010) and Asparouhov and Muthén (2014). This approach is a commonly used multi-step method for estimating mixture models that incorporate auxiliary variables, such as covariates and distal outcomes. Including these auxiliary variables helps avoid unintended measurement model changes (Vermunt, 2010). In our context, we specify the regression relationship for each latent class rather than simply including covariates or distal outcomes.

Detailed discussions of the ML three-step procedure can be found in various papers, including Vermunt (2010) and Asparouhov and Muthén (2014). Specific Mplus (Muthén & Muthén, 1998–2021) syntax for specifying this model is provided in Nylund-Gibson et al. (2014, 2022). We also included detailed R code (R Core Team, 2025) and annotated output for our example in text and in the supplemental materials https://github.com/dinanajiarch/lca_moderation. Specifically, Appendix A contains the R code using the MplusAutomation package (Hallquist & Wiley, 2018), and Appendix B provides the accompanying annotated output. The ML three-step procedure for a latent class moderation model can be summarized as follows:

- Step 1: After performing class enumeration to determine the optimal number of classes, estimate the unconditional latent class model, independent of the predictors and distal outcomes, and save each individual’s modal class assignment (their most likely latent class) and the logits for the classification probabilities.
- Step 2: Extract the modal class assignment (most likely latent class) and the logits from Step 1 to account for measurement error in the modal class assignments.
- Step 3: Specify the latent class moderation model with auxiliary variables. The modal class assignment is used as an indicator of latent class membership, with parameters fixed at the Step 2 logits to account for measurement error. To specify a moderation model, a linear regression of the distal outcome(s) on the predictor(s)/covariates(s) is freely estimated across each latent class.

### Testing for moderation

After specifying the ML three-step, moderation is tested by evaluating differences in the slope and intercept across latent classes. The stages of testing for moderation are described in detail in Table 2, which includes specifying tests for moderation in Mplus and interpreting the results. To avoid confusion with the ML three-step procedure, we refer to the moderation testing steps as stages (i.e., Stage I, Stage II, and Stage III). The slope and intercept tests focus on evaluating moderation and consist of the first two stages:

(I) An omnibus Wald test to assess overall differences across classes, and
(II) Pairwise comparisons conducted only if the omnibus test is significant.

**Table 2:** Stages of analyses for slope and intercept estimation in a latent class moderation

Test	Stage	Test	Mplus syntax	Result
Slope	I	Wald test: Does the relation (slope) differ across the latent classes? β_11 =_β_12 =…=_β_1K_	MODEL TEST: !Wald Test for Slopes B11=B12; B12=B13;	If significant, we have evidence of moderation and interaction. Proceed to stage II to test for pairwise differences.
	II	Pairwise differences: Where are the slope differences? Is β_11 =_β_12_? Is β_11 =_β_13_?	MODEL CONSTRAINT: new (slope12, slope13, slope23); slope12=b11-b12; slope13=b11-b13; slope23=b12-b13;	If significant, there is a slope difference between the two classes examined.
Intercept	I	Wald test: Are the distal means (intercept) different across the latent classes? β_01 =_ β_02=…=_ β_0K_	MODEL TEST: !Wald Test for Intercepts B01=B02; B02=B03;	If significant, we have evidence of distal mean differences. Proceed to stage II to test for pairwise differences.
	II	Pairwise differences: Where are the pairwise differences? Is β_01 =_ β_02_? Is β_01 =_ β_03_?	MODEL CONSTRAINT: new (int12, int13, int23); int12=b01-b02; int13=b01-b03; int23=b02-b03;	If significant, there is an intercept difference between the two classes examined.
Within-class regression	III	Is the slope for a specific class significantly different than zero? β_1c_ = 0?	Exists in the class-specific statements. Example: MODEL: %C#1% [DISTAL] (B01); DISTAL; **DISTAL on** **PREDICTOR(B11);**	If significant, the slope in the class examined significantly differs from zero.
	III	Is the intercept for a specific class significantly different than zero? β_0c_ = 0?	Exists in the class-specific statements. Example: MODEL: %C#1% **[DISTAL] (B01);** DISTAL; DISTAL on PREDICTOR(B11);	If significant, the intercept in the class examined significantly differs from zero.

In addition to testing for moderation, the model also estimates within-class regression slope and intercept coefficients (Stage III).

#### Slope differences

To test for moderation or the equivalence of the slopes across the latent classes, we use the omnibus Wald chi-square test (Stage I). Conceptually, a Wald test tests if there is evidence of a significant relation between the latent class variable and the slopes. In this context, we set the slopes equal to each other. If this test is significant, we conclude that there is evidence that at least one slope is significantly different (similar to an overall *F*-test in analysis of variance [ANOVA]). We conduct pairwise differences to evaluate further which class-specific slopes are significantly different (Stage II).

#### Intercept differences

Additionally, we can test the equivalence of the regression intercepts or the mean of the distal outcome variable using the Wald chi-square test. Similarly, this tests if there is a relation between the latent class variable and the intercepts (Stage I). If there is evidence of a difference, then the pairwise comparisons of the intercepts are completed (Stage II).

#### Within-class regressions

While this is not evidence of moderation, we can examine individual regression slopes and intercepts across classes and report whether the estimates for a specific class are significantly different from zero (Stage III). These class-specific slopes and intercepts can be interpreted to provide a more nuanced understanding of the model and better understand the significance of the predictor-outcome relationship within each class.

### Data visualization

To visually display evidence of moderation (significant differences in slopes) and mean differences (significant differences in intercepts), a table of slope and intercept values across latent classes and their significance, as well as a simple slopes graph and distal means bar chart, are recommended and is illustrated in this paper using an applied example on science attitudes.

#### Example: Heterogeneity in science attitudes

To illustrate moderation with a latent class variable, we examine heterogeneity in 12th-grade level students’ attitudes towards science in relation to their science achievement and interest in science issues. While a relatively small percentage of students will pursue a career in a STEM field, having an interest in science-related issues impacts all students. For example, not all students will pursue an advanced degree to develop low-cost solar cell applications from inorganic materials. However, all students have opportunities to make decisions related to the environment, such as voting to subsidize solar energy technologies.

Recognizing that students with high achievement are not the only ones interested in science issues, research suggests that other factors, such as attitudes towards science, can contribute to interest in science issues. Students may have low achievement in science and be supportive of science-related issues. Science attitudes may influence this relationship between achievement and support of science issues. Osborne and colleagues' (2003) literature review suggests that attitudes toward science are not a unitary construct. Attitudes may include components such as self-efficacy in science and perceptions of science teachers. Despite the range of ways attitudes have been operationalized in the literature, there is a general consensus that attitudes can impact students’ interest in science issues.

To capture the complexity of attitudes toward science, we employ latent class analyses (Ing & Nylund-Gibson, 2013, 2017) within our moderation model. Additionally, we recognize the importance of considering demographic characteristics, such as race and gender, which have been shown to influence disparities in science opportunities and outcomes (Bottia et al., 2021; Riegle-Crumb et al., 2019; Saw et al., 2018). We aim to model more nuanced relationships between complex phenomena by incorporating these variables into our moderation models.

#### The focus of this tutorial: Latent class moderation in Mplus

Using an applied example, this paper illustrates the steps in fitting a latent class moderation model, interpretation, and visualization. This tutorial describes moderation with latent class analysis using the ML three-step method with auxiliary variables (Asparouhov & Muthén, 2014), as implemented in Mplus via the MplusAutomation (Hallquist & Wiley, 2018) package. This package is a free R wrapper that facilitates the automation of latent variable models in Mplus. The modeling process was conducted within RStudio, which provided a convenient platform for documenting the workflow and adding explanatory comments. Additionally, Mplus syntax and suggested tables and figures are provided to aid the interpretation and presentation of results.

## Method

### Data source

The dataset used in this example comes from the Longitudinal Study of American Life (LSAL; Miller, 2010), funded by the National Science Foundation (NSF) in 1986. The LSAL was designed to study the development of student achievement in middle and high school, as well as the relationship between these patterns and later career choices. The LSAL used telephone interviews and questionnaires to survey the two cohorts (younger and older) of seventh graders every year for 7 years in school (*n* = 5,945). The demographics in the final sample were predominantly white (74%), with an approximately equal number of females (49%) and males (51%). About 58% of the students’ mothers have at least a high school diploma, 10% have some college education, and 11% have a 4-year college degree. This study focused on a subset of the total sample to reflect survey responses from 12th-grade students (*n* = 3,019 students).

### Measures

The latent class variable was used as a moderator to examine the relationship between science achievement (predictor) and interest in science issues (distal outcome). We then linked these classes to the covariates (gender, ethnicity, and socioeconomic status).

#### Latent class variable

Science attitude items for the latent class variable were selected based on prior research using the same data (Ing & Nylund-Gibson, 2013, 2017). The items chosen reflect a social cognitive career theory perspective that highlights the students’ self-efficacy, outcome expectancies, and personal goals (Lent et al., 1994). The questions have the same response options on a Likert-type scale (strongly agree, agree, not sure, disagree, strongly disagree). Additionally, the items were dichotomized; strongly agree and agree were coded as “1,” and disagree/strongly disagree/not sure were coded as “0.” The response options are used so that a “1” represents endorsing the science attitude, and a “0” represents that a student did not endorse that item.

#### Auxiliary variables

##### Distal outcome

Students responded to four items that asked about their interest in science issues in 12th grade. They rated their interest in particular science-related issues on a three-point scale: Not at all interested, moderately interested, and very interested. Four science-related issues were chosen for analysis: space exploration, scientific discoveries, inventions/technologies, and energy policy issues. The items were used to create a latent factor of students’ interest in science issues. A factor, as opposed to a composite variable, was used as a distal outcome to reduce measurement error bias and provide the ability to look at the validity of the items using model fit indices. The overall goodness of fit indices in the final factor analysis model of the four items suggest the model fit the data well: χ^2^(2) = 30.112, *p* <.001, SRMR =.014, RMSEA =.063 (90% CI =.045 –.084), CFI =.99, TLI =.97. For identification in the moderation model, the factor means for one of the classes was fixed to zero, similar to what is done when exploring measurement invariance across groups (van de Schoot et al., 2012).

##### Predictor

Science item-response theory (IRT) achievement scores measured at the 11th grade was used as a predictor.

##### Covariates

Gender, ethnicity, and socioeconomic status were used as covariates. Gender and ethnicity are treated as dichotomous variables. Ethnicity was dichotomized to represent students typically represented (e.g., Asian and White) and those typically underrepresented in STEM fields (e.g., African American, Hispanic, Native American). For ethnicity, or the underrepresented minority (URM) variable, “0” represents students who are typically represented in STEM fields, and “1” denotes students who are typically underrepresented in STEM fields. For gender, “0” represents males, and “1” represents females. The mother’s educational attainment was a proxy for socioeconomic status (SES). The original variable in the LSAL dataset consisted of nine levels: less than high school, high school graduation only, vocational or trade school, some college, associate degree, bachelor’s degree, master’s degree, PhD/MD, or “I don’t know”. The variable was collapsed for this study to include only five levels of education: less than high school, high school diploma, some college, 4-year college, and an advanced degree.

### Moderation with latent class analysis

**Fig. 2:**
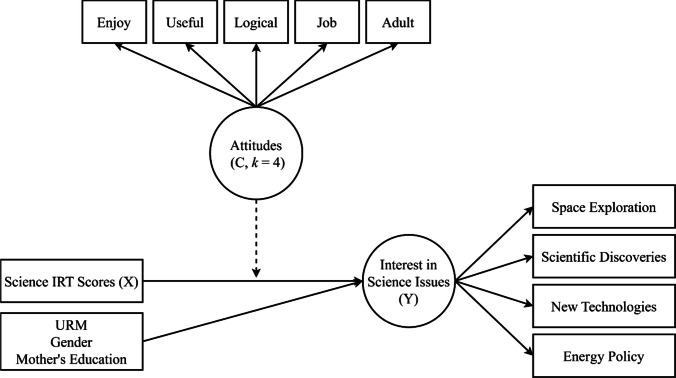
Path diagram: relationship between science ability and issues in science moderated by latent class attitudes variable. *Note*. The dotted arrow from the latent class variable *C* to the regression of outcome *Y* on predictor *X* indicates that the slope and intercept vary across the latent classes

The specification of moderation using a latent class variable is conducted in several steps, delineated here. The path diagram of the hypothesized model in Fig. 2 is similar to moderation using linear regression, where the moderator (latent class variable) is hypothesized to moderate the relation between the predictor (science ability scores at 11th grade) and the outcome (interest in science issues at 12th grade), controlling for the demographic variables.

#### Class enumeration

First, class enumeration for the latent class variable using the five science attitude indicators is conducted using recommended approaches to enumeration without the auxiliary variables (Nylund-Gibson & Choi, 2018; Nylund-Gibson & Masyn, 2016). Multiple indicators of model fit were used to determine the final number of classes: consistent Akaike’s information criterion (CAIC), Bayesian information criterion (BIC), adjusted Bayesian information criterion (aBIC), approximate weight of evidence criterion (AWE), Vuong-Lo-Mendell-Rubin likelihood ratio test (VLMR-LRT), and bootstrapped likelihood ratio test (BLRT). Lower BIC, CAIC, aBIC, and AWE values indicate a better-fitting model (Masyn, 2013). For the BLRT and VLMR-LRT,* p*-values less than 0.05 indicate that the model has not significantly improved compared to the model with one less class (Nylund et al., 2007).

#### Model estimation

All models were fitted using the Mplus software package through the MplusAutomation. Detailed code, annotated output, and workflow documentation for the example presented in this paper can be found in text and at https://github.com/dinanajiarch/lca_moderation. Regarding estimation techniques, models were estimated using maximum likelihood (ML) estimation under the missing at random (MAR) assumption. We utilized random starts during the enumeration phase, minimizing the risk of converging to a local solution rather than a global one.

After deciding on the latent class solution, the latent class moderation model was estimated using the ML three-step procedure described above (Vermunt, 2010). A walkthrough is discussed in detail, accompanied by R code and Mplus syntax, in the results section.

#### Testing for moderation

To test for moderation, we simultaneously estimated the latent class variable and allowed the relationship between the predictor variable (science ability) and the distal outcome to vary across the latent classes while holding the measurement of the latent class variable constant. This was achieved using the ML three-step procedure. The predictor variable, science ability, was scaled and centered. For comparison and identification, the factor means for one of the classes, in this example, the second class (ambivalent with minimal utility), was fixed to zero, similar to what is done in the context of factor mean comparison in measurement invariance. Alternatively, the researcher could swap out the factor's mean to be zero of a different class if they are interested in other comparisons.

## Results

This section presents the implementation and results of the moderation example using a latent class variable. We provide the Mplus syntax implemented through the “MplusAutomation” package in R. A walkthrough of R code and model output is included to illustrate each step of the analysis.

### Descriptive statistics

Descriptive statistics were calculated using both base R functions and Mplus through the MplusAutomation package. R code and Mplus syntax for estimating descriptive statistics in MplusAutomation are presented below:
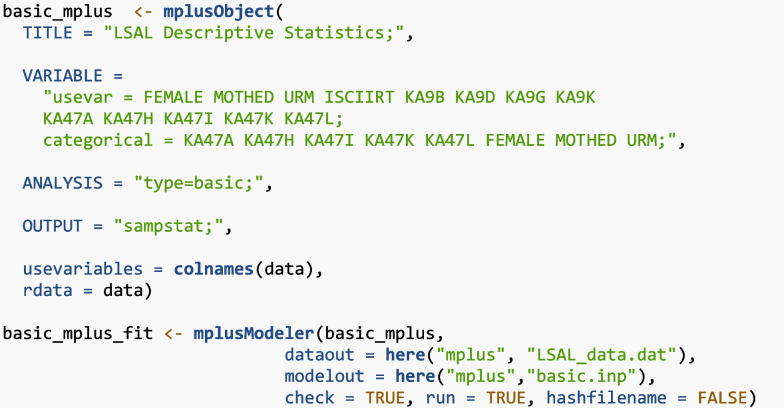


The “mplusObject” function is used to generate the Mplus model object in R, stored here as “basic_mplus”. This function specifies Mplus commands (e.g., TITLE, VARIABLE, ANALYSIS, OUTPUT) as arguments, with Mplus syntax used in quotes within each command. Within each command, options can be specified, and each option must end with a semicolon. For example, in the VARIABLE argument, there are two Mplus options (“usevar” and “categorical”). All variables that were included in the analysis are listed under “usevar”, and categorical variables were specified under “categorical.” Variable names must match the R dataset (in this case, the data is stored as “data”). In the ANALYSIS command, “type = basic” is used to request a basic analysis, and in the OUTPUT command, “sampstat” is used to display sample statistics in the output file. In addition to the Mplus-specific arguments, the “mplusObject” function also includes R-specific arguments, such as “rdata” (which provides the dataset stored in R) and “usevariables” (which lists the variable names in the entire dataset).

The model is then estimated using the “mplusModeler” function, which estimates the model and saves three files to the computer: the input file (.inp), the output file (.out), and the dataset (.dat). The “dataout” argument specifies the name and location to save the dataset, and the “modelout” argument specifies the name and location for the input and output files. The “here” function is used to create a file path starting at the top-level directory of your R project. Additionally, “check=TRUE” checks for missing semicolons, “run=TRUE” runs the model, and “hashfilename=FALSE” does not add a hash of the raw data to the datafile name.

Below is a snippet of the “type=basic” output, where ISCIIRT is the science IRT score variable and KA9B–KA9K are the “interest in science issues” variables:
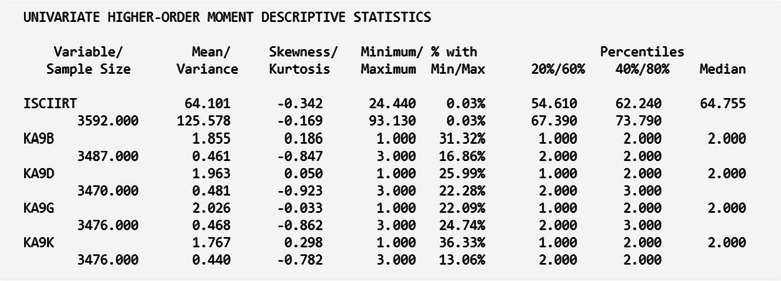


After running “mplusModeler”, the “get_sampstat” function from the MplusAutomation package can be used to extract the descriptive statistics output into R.
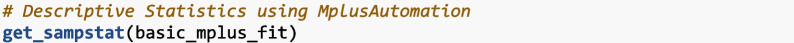


In contrast, using base R, the summary function can be applied directly to the data object. Although generating descriptive statistics through MplusAutomation requires more setup than using summary in base R, it provides much more detailed information. Specifically, the summary function in R only displays basic quartiles, whereas MplusAutomation provides correlations, proportions, means, variances, skewness, kurtosis, and a summary of missing data patterns.
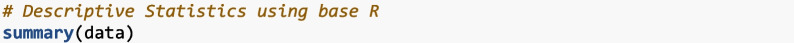


Table 3 presents the endorsement proportion for the five latent class indicators (science attitudes) and the means and standard deviations for the predictor (science IRT scores) and distal outcome measures (interest in science issues). Overall, the endorsement of each item varied, with the least endorsed item at 34% and the highest endorsed item at 55%. The latent class indicator most endorsed by students was the “Science Helps Logical Thinking” item. The second most endorsed item was “I Enjoy Science,” with 53% endorsement. Conversely, the least endorsed item is “Need Science for a Good Job,” with 34% endorsement. The average score of science ability is about 64, ranging from 24 to 93.

**Table 3:** Descriptive statistics for latent class indicators and auxiliary variables

	Item label	Endorsement proportion or mean (*SD*)	*n*
*Latent class indicators*			
	I Enjoy Science	.53	1,793
	Science is Useful in Everyday Problems	.45	1,502
	Science Helps Logical Thinking	.55	1,825
	Need Science for a Good Job	.34	1,139
	Will Use Science Often as an Adult	.40	1,352
*Predictor*			
	11th grade science IRT score*	64.10 (11.21)	2,672
*Distal outcome* (Interest in Science Issues)			
	Space Exploration	1.87 (0.67)	2,651
	Scientific Discoveries	1.98 (0.69)	2,639
	New Technologies	2.04 (0.68)	2,643
	Energy Policy Issues	1.76 (0.66)	2,647

^*^ Uncentered mean and standard deviation.

### Class enumeration

A series of LCA models were fit, starting with a one-class model until non-convergence was achieved (one- to six-class models). The following chunk of R code shows how to estimate multiple Mplus models using MplusAutomation with base R’s “lapply” function:
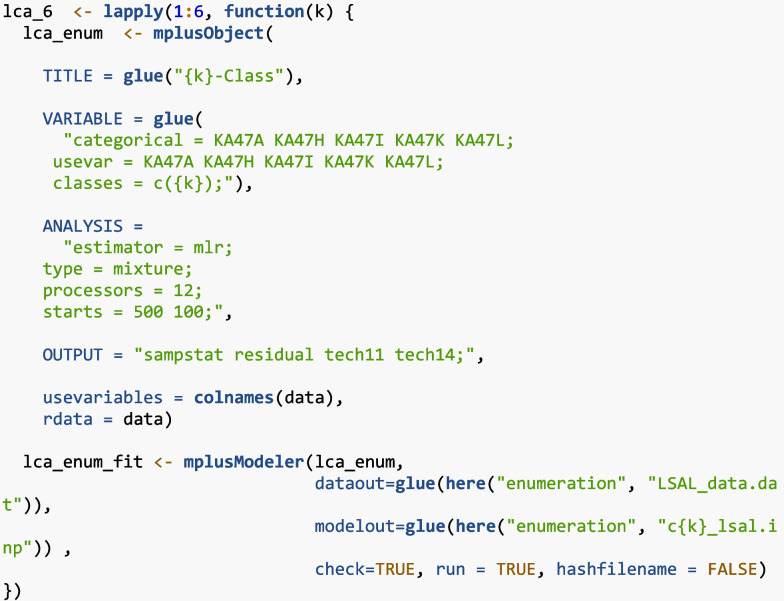


The “lapply” function iterates over multiple latent class models, *k*, specifying one through six classes, as specified by 1**:**6. The “glue” function is used to dynamically generate model syntax that is enclosed by braces to be evaluated as R code. For example, the {*k*} in “classes =c({k})” will be replaced with 1 to specify the 1-class model first, then loop through the rest of the class solutions and stop after the six-class model is estimated. An “mplusObject” model was created for each model, which included the set of science attitude indicators from the LSAL dataset (KA47A, KA47H, KA47I, KA47K, KA47L). All models were estimated using the maximum likelihood with robust standard errors (“estimator = MLR”) as a mixture model (“type = mixture”). To ensure a stable solution, each model used 500 random sets of starting values with 100 final optimizations (“starts = 500 100”). Model output included descriptive statistics (“sampstat”), residuals (“residual”), and the likelihood ratio tests, BLRT (“tech14”) and VLMR (“tech11”) to help determine the number of classes. The models were estimated in Mplus via the “mplusModeler” function, which also saved the input and output files to a designated folder for each class solution.

After estimating the latent class models, it is important to review each Mplus output file to ensure that the models were estimated normally and properly identified. In the LSAL example, the six-class model was not identified and excluded from evaluation. After reviewing the model outputs, model enumeration results were extracted using “readModels” from the MplusAutomation package. This imports the full model output for each class into R as a structured list. Then, we summarized key fit indices using “LatexSummaryTable”. Additional fit indices (e.g., CAIC and AWE) were computed manually as they are not included in the Mplus output. A simplified code snippet is shown below. Full APA formatting using the “gt” package is provided in Appendix A.
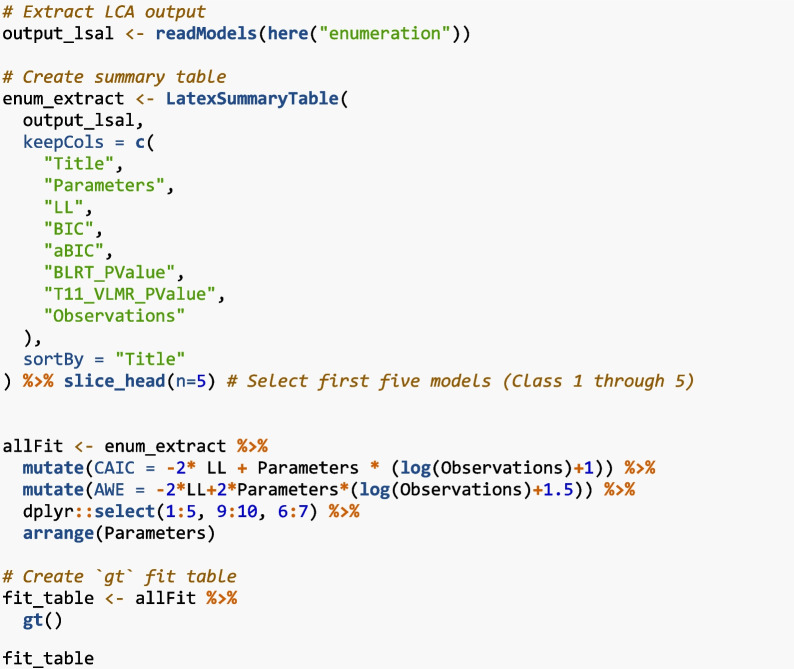


Table 4 presents the fit indices that compare the models with one through six latent classes. The information criteria (BIC, aBIC, CAIC, and AWE) reached a minimum value for the four-class model. Likelihood ratio tests did not produce nonsignificant *p*-values and did not point to a specific best-fitting model. Since four of the six fit indices suggest the four-class model, the four-class model was retained to describe the heterogeneity of science attitudes in 12th-grade students.

**Table 4:** Fit statistics for class enumeration for 12th grade science attitudes

Model	*K*	LL	BIC	aBIC	CAIC	AWE	BLRT *p*	VLMR *p*
*n* =3,364	1	−11,315.87	22,672.34	22,656.45	22,677.34	22,727.94	-	-
	2	−9,009.08	18,107.48	18,072.53	18,118.48	18,229.81	<.001	<.001
	3	−8,814.56	17,767.18	17,713.17	17,784.18	17,956.24	<.001	<.001
	4	−8,742.24	**17,671.26**	**17,598.17**	**17,694.26**	**17,927.04**	<.001	<.001
	5	−8,734.82	17,705.15	17,613.01	17,734.15	18,027.66	<.001	<.001

K = number of classes; LL = model log-likelihood; BIC = Bayesian information criterion; aBIC = sample size adjusted BIC; CAIC = consistent Akaike information criterion; AWE = approximate weight of evidence criterion; BLRT = bootstrapped likelihood ratio test; VLMR-LRT = Vuong-Lo-Mendell-Rubin adjusted likelihood ratio test; *p* = *p*-value; **Bold** = best fit statistic for each individual statistic

**Fig. 3:**
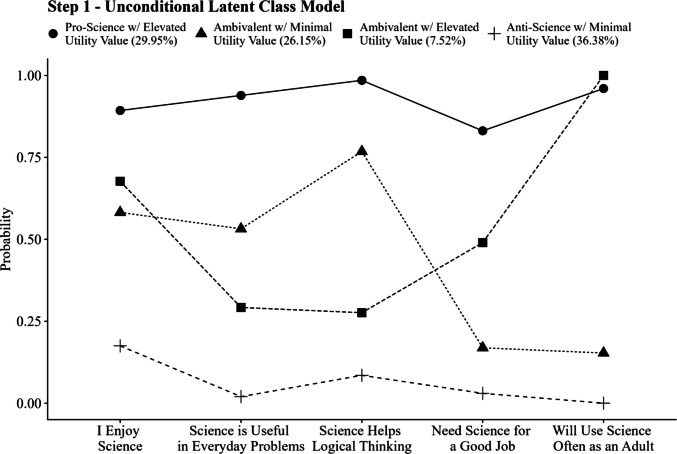
Proportions of 12th graders endorsing each item by attitudinal class


After choosing the four-class model, the conditional item probability plot is used to interpret and label the four emergent latent classes (Fig. 3). A separate code file for generating the conditional item probability plot is included in Appendix A. The code below demonstrates how to generate the plot using the saved model output:

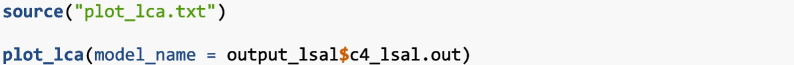


The classes were labeled *Pro-Science with Elevated Utility* (i.e., high probability of endorsement for all items), *Ambivalent with Minimal Utility* (i.e., moderate endorsement probability for the three items of “I Enjoy Science,” “Science is Useful,” and “Science Helps Logical Thinking,” with a lower probability of endorsement for the items “Need Science for a Good Job” and “Will Use Science Often as an Adult”), *Ambivalent with Elevated Utility* (i.e., moderate endorsement probability for the three items “Science is Useful,” “Science Helps Logical Thinking,” and “Need Science for a Good Job,” with a higher probability of endorsement for the items “I Enjoy Science” and “Will Use Science Often as an Adult”), and *Anti-Science with Minimal Utility* (i.e., low probability of endorsement for all items). The *Pro-Science with Elevated Utility* class makes up 29.95% of the sample, *Ambivalent with Minimal Utility* consists of 26.15% of the sample, *Ambivalent with Elevated Utility* consists of 7.52% of the sample, and *Anti-Science with Minimal Utility* makes up 36.38% of the sample.

### Test for moderating effect of science attitudes

To test for moderation, we used the ML three-step method to estimate the regression model in which the intercept and slope of a linear relationship between predictors and a distal outcome could vary across latent classes. Classes of science attitudes (*C*) were tested as a moderator of the relationship between science scores (e.g., ISCIIRT) and interest in science issues (e.g., ISSUES).

#### ML three-step method

After completing latent class enumeration and identifying the optimal latent class solution, Step 1 of the ML three-step process involves estimating the unconditional latent class model, independent of the predictors and distal outcomes. Then, Step 2 saves each individual’s modal class assignment (i.e., their most likely latent class) and the logits for the classification probabilities. Finally, the Step 3 specifies the overall structural model using the modal class assignment as the latent class variable and the logits to account for measurement error.

#### Step 1

To complete the ML three-step method with MplusAutomation in R, Step 1 specifies the latent class indicators in the “usevar” and “categorical” options, four classes with “classes = c(4)”, and includes all predictors and distal outcomes in the “auxiliary” options. Under the ANALYSIS command, take the optimal seed (“optseed = 573096”) in the four-class output created during enumeration to replicate the solution. This value can be found in the final stage log likelihood value table in the four-class output. Under the SAVEDATA command, use “save=cprob” to save the individual posterior probabilities for each latent class and the modal class assignment (along with the other analysis variables), the file name is saved under “file = savedata.dat”. The option “format = free” formats the file delimited by a space.


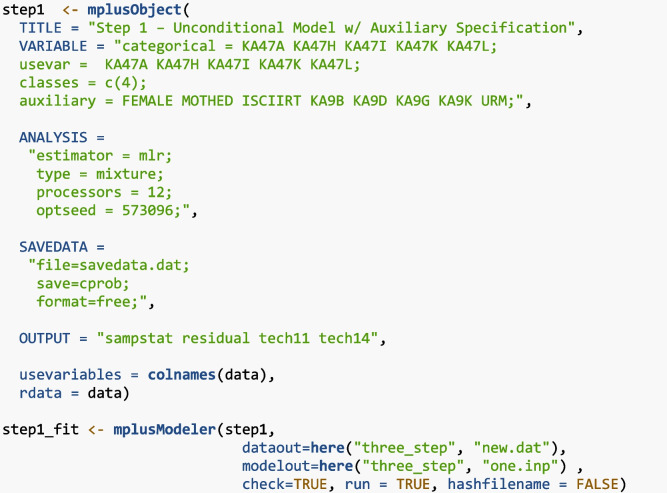


Below are the logits for the classification probabilities for the most likely latent class membership by latent class presented in the Step 1 output:


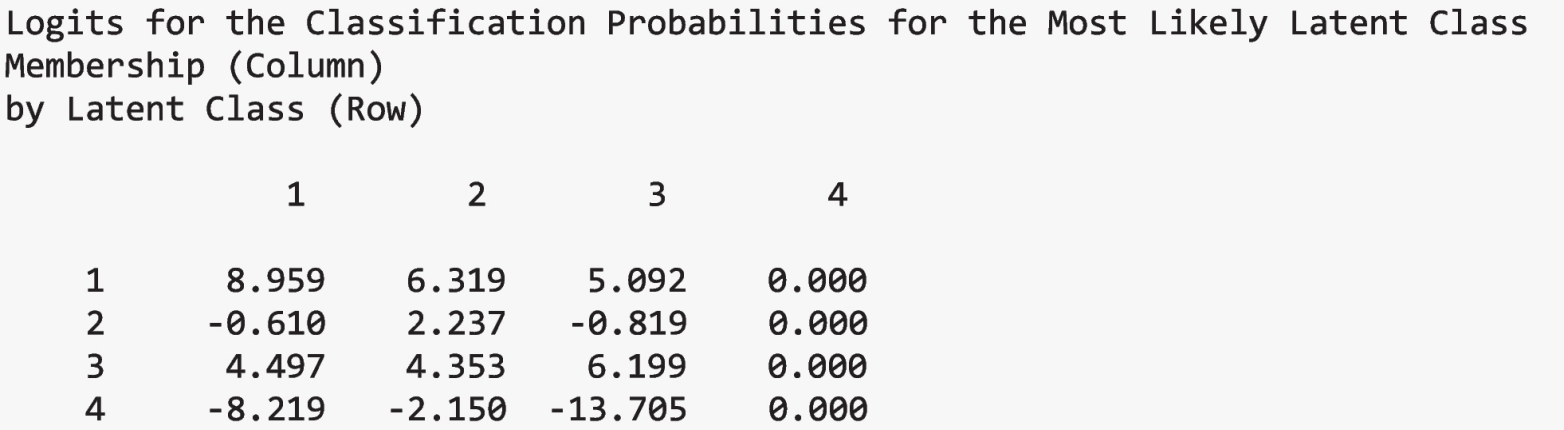


#### Step 2

Step 2 extracts the modal class assignment and the logits from the output and data created in Step 1. Below is the code to complete this step in R. The first line reads in the Step 1 .out file using “readModels”. The second line extracts the logit values for the most likely class from the “output_one” object. In the third line, the dataset created in Step 1 is extracted, which includes each individual’s modal class assignment and auxiliary variables. Finally, the column “C” (automatically assigned by Mplus to represent class membership) in the dataset is renamed to “N”. This renaming step is optional but helps maintain consistency when referencing the nominal latent class variable in Step 3.


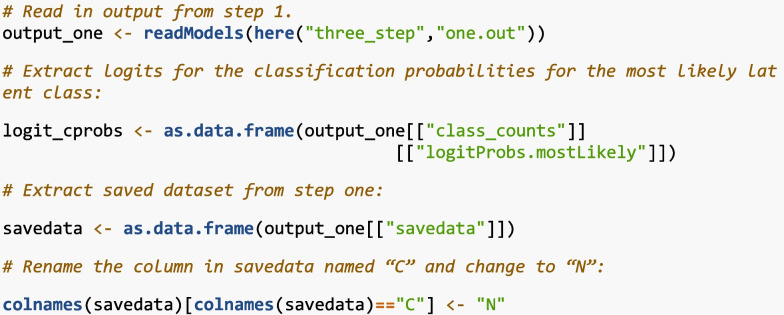


#### Step 3

In Step 3 of the ML three-step, a structural LCA model that incorporates the auxiliary variables is specified. The modal class assignment is used as a nominal indicator of latent class membership, and the classification logits are fixed in the model to account for measurement error in modal class assignment. Because the latent class variable is categorical, a multinomial logistic latent class moderation model was used to assess the relationships between the covariates and the latent class.

To test for moderation, a new Mplus model is created with “mplusObject”. Included in the “usevar” option are the auxiliary variables, or the covariates (gender, mother’s education, and URM), the predictor (Science IRT scores), the distal outcome factor indicators (KA9B, KA9D, KA9G, KA9K), and the nominal modal class assignment “N”. The ISCIIRT variable was scaled by dividing by 10 and grand-mean centered using the DEFINE command in Mplus. The MODEL command defines the structural relationship in the latent class moderation model. The %OVERALL% section specifies the model parameters that are held constant across all classes. First, we specify the factor model ISSUES using the “by” statement, which means the factor ISSUES is measured by four observed items (KA9B, KA9D, KA9G, KA9K). The second line (ISSUES on FEMALE MOTHED URM) regresses the latent factor on the covariates. The third line (ISSUES on ISCIIRT) regresses the distal outcome, ISSUES, on the science IRT scores (ISCIIRT).

The following describes the class-specific regressions. For Class 1, referred to as %C#1% in Mplus syntax, we use “glue” and the “logit_cprobs” object to insert the classification logits from the first row in the matrix in order to adjust for measurement error in the modal class assignment, N. The statement [ISSUES] (B01) estimates the intercept (mean) of the latent factor (ISSUES) in Class 1 and is labelled “B01” to use in later sections. Next, ISSUES (without brackets) estimates the residual variance of the latent factor in Class 1. Finally, and most relevant to the moderation model, ISSUES on ISCIIRT (B11) estimates the class-specific slope of the science IRT scores predicting the latent factor (interest in science issues). This syntax is repeated for each subsequent class. The intercept for the second class, which we determined to be an appropriate reference class, is set to zero for model identification purposes.

The next two Mplus commands (MODELTEST and MODELCONSTRAINT) are used to conduct an omnibus Wald test and pairwise comparisons, respectively. Both formally test whether regression parameters, specifically slopes and intercepts, significantly differ across classes. The MODELTEST command estimates a Wald chi-square test to evaluate whether a set of parameters are jointly equal across classes (e.g., groupwise test). A limitation of the way the Wald test is specified in Mplus is that only one Wald test can be specified at a time. In this example, the second omnibus test (for intercepts) is commented out using an exclamation point.

For the first omnibus test, the class-specific slopes are tested to see if there is a relation between the latent class variable and slope parameters by testing if the slope parameters are equal across all classes. The second Wald test assesses whether the intercepts are the same across classes (except for the second class, which is fixed at zero). If there is evidence of differences in either parameter (intercept and slope), then pairwise comparisons are explored. The MODELCONSTRAINT command tests the pairwise differences in the slopes and intercepts across classes, using the NEW option to label each pair. A set of new parameters is created, one for each pairwise comparison. For example, to compare the estimated slope parameter of Class 1 and Class 2, we create a new variable, “SLOPE12”, which is defined as “slope12=B11-B12”. If SLOPE12 is not significant, then we conclude that there is not a significant difference between the slope of Class 1 (B11) and Class 2 (B12). This is done for all pairwise comparisons of the slopes and similarly for the distal outcome means. In terms of process, first test for differences in the parameters across the latent classes using the Wald test (Stage I). If significant, then examine the pairwise differences to explore where these differences appear (Stage II). Appendix A contains additional code for generating tables and figures related to the latent class moderation results.


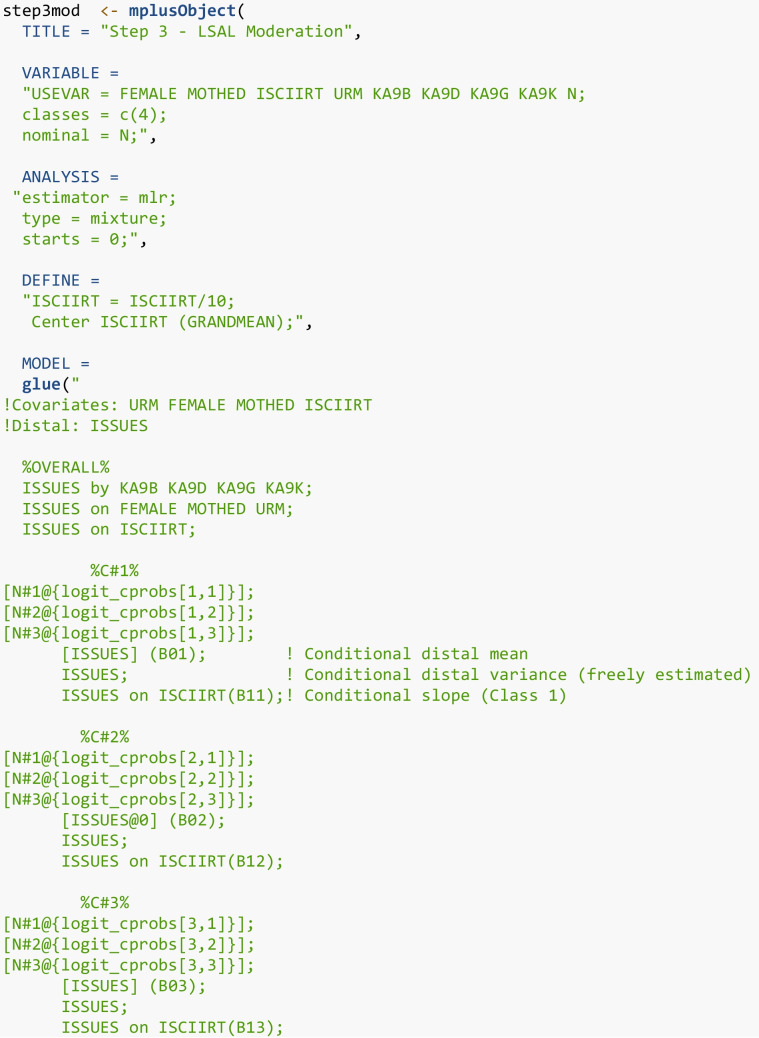

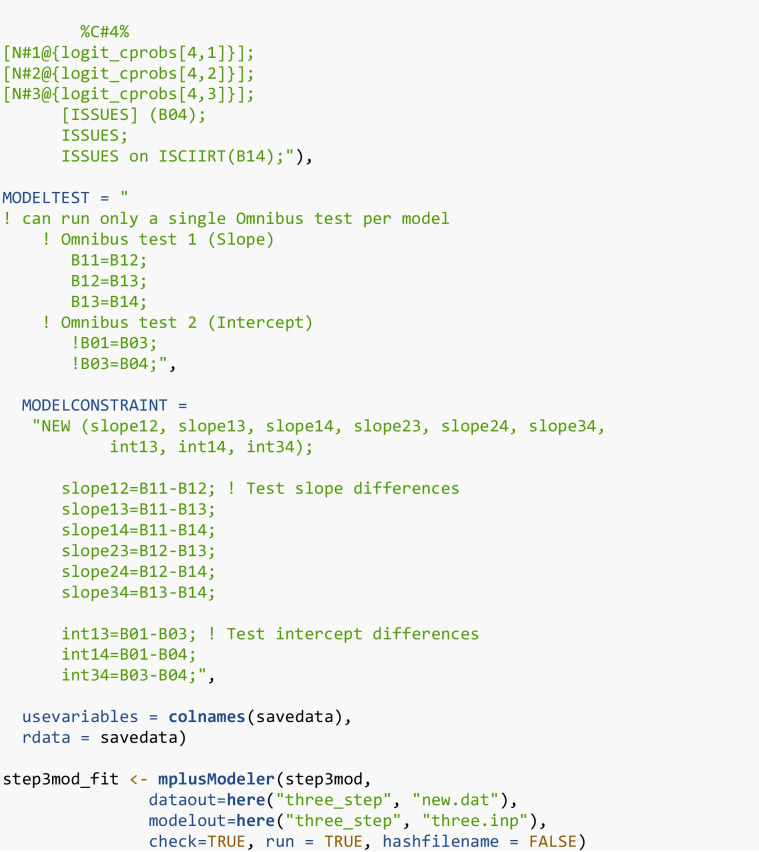


#### Moderation results

The order of results corresponds to Table 2. Annotated Mplus output is presented in each section below. All output shown is from the same “three.out” file created in the previous section (except for the second Wald test, which must be rerun to obtain the test results for slopes and intercepts, separately). Additional annotated output is included in Appendix B of the supplemental materials, which provides a detailed walkthrough of each section of the latent class moderation model. The latent class labels used in the output are as follows:

1. *Pro-Science with Elevated Utility Value*
2. *Ambivalent with Minimal Utility Value*
3. *Ambivalent with Elevated Utility Value*
4. *Anti-Science with Minimal Utility Value*

**Fig. 4:**
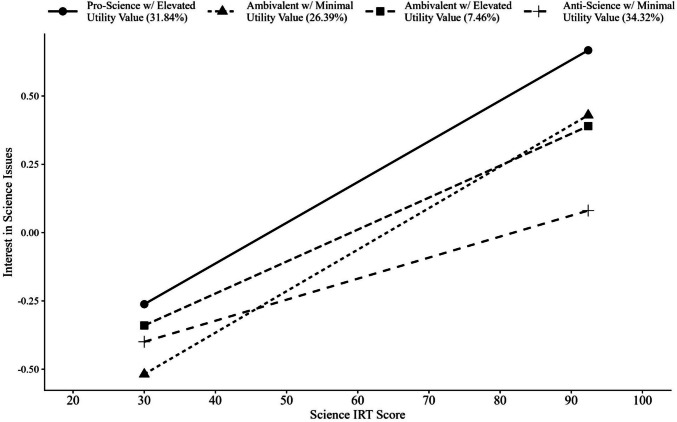
Simple slopes graph of science ability predicted by issues in science (uncentered)

**Fig. 5:**
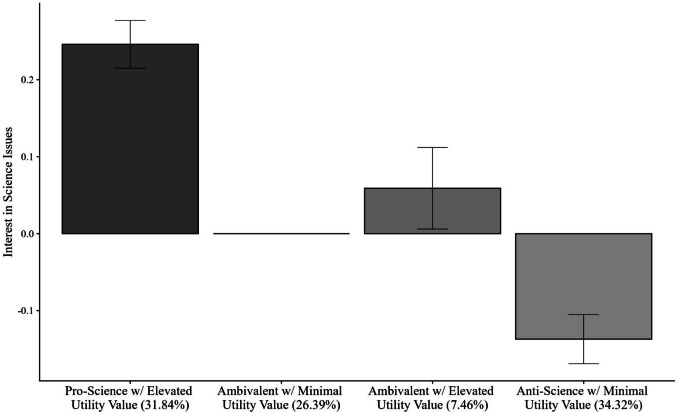
Means of interest in science issues across classes of science attitudes

Figures 4 and 5 present the relationships between the slope and intercept parameters estimated independently for each latent class. In this graph, the *x*-axis is fixed to be uncentered and unscaled. Table 5 presents a table of the slope and intercept values across science attitude classes.

**Table 5:** Slope and intercept values across classes of science attitudes

		Science ability 11th grade (X)		➔	Interest in science issues 12^th^ grade (Y)	
		(*Slope*)			(*Intercept*)	
*k*	Class label	Estimate (*se*)	Sig. class differences?		Estimate (*se*)	Sig. class differences?
C1 _*n* = 815_	*Pro-Science w/Elevated Utility Value*	0.149 (.015) **	C4		0.264 (.031) **	C4, C3
C2 _*n* = 733_	*Ambivalent w/Minimal Utility Value*	0.152 (.023) **	C1		0^†^	None
C3 _*n* = 186_	*Ambivalent w/Elevated Utility Value*	0.117 (.033) **	None		0.059 (.053)	C1, C4
C4 _*n* = 857_	*Anti-science w/Minimal Utility Value*	0.077 (.018) **	C2, C1		−0.137 (.032) **	C3, C1

The Science ability column represents slope values, and the Interest in science issues column represents intercept or mean values, *p <.05. **p <.01

^†^ Intercept and *se* for C2 are not estimated because the mean is fixed to zero to identify the latent factor

#### Slope differences

There is evidence of a significant moderation (Stage I: Wald Test χ^2^(3) = 11.003, *p* =.012). That is, there is a statistically different relationship between the predictor (science achievement scores) and the distal outcome (interest in science issues) across at least one of the classes.
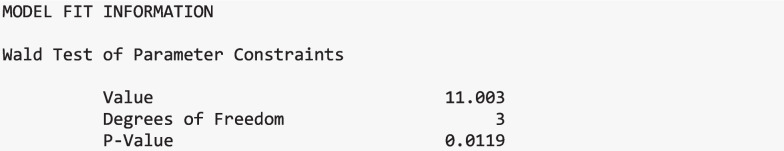


##### Pairwise slope differences

To further investigate which class-specific relations differ, pairwise comparisons of the regression slopes on the distal outcome, issues in science, were completed (Stage II). When examining the pairwise slope differences for interest in science issues regressed on science achievement, the *Anti-Science with Minimal Utility* class significantly differed from the *Ambivalent with Minimal Utility* and *Pro-Science with Elevated Utility* classes, *p* = 0.019 and *p* = 0.001, respectively. Specifically, the rate at which science achievement predicts interest in science issues differs among these classes. Figure 4 visually presents the relations between science achievement and interest in science issues across each class. There were no other significant slope differences across classes.


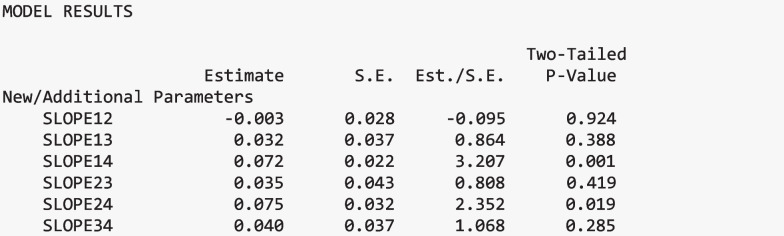


#### Intercept differences

There was evidence of significant differences in the distal outcome means, or the intercepts, across the science attitude classes, Stage I: Wald Test χ^2^(2) = 205.615, *p* <.001. This suggests that at least one pairwise difference of the distal outcomes means is significant across classes. Figure 5 presents the means of the science achievement (grand mean-centered and scaled) across the classes of science attitudes.
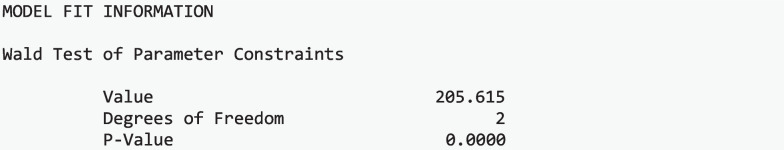


##### Pairwise distal outcome differences

To further investigate which class-specific relations differ, pairwise comparisons of the distal outcome across each class were studied (Stage II). Pairwise tests found significant differences between *Pro-Science with Elevated Utility* class and *Ambivalent with Elevated Utility* class, *p* <.001, and *Anti-Science with Minimal Utility* class, *p* <.001. Additionally, there was a statistically significant difference between the *Ambivalent with Elevated Utility* class and the *Anti-Science with Minimal Utility* class, *p* <.001. This implies that the two classes, on average, have significantly different interests in science issues. Specifically, those in *the Pro-Science with Elevated Utility* class have higher interests in science than those in the *Anti-Science with Minimal Utility* and *Ambivalent with Elevated Utility* classes. Additionally, those in the *Ambivalent with Elevated* Utility class have higher interests in science than those in the *Anti-Science with Minimal Utility* class.


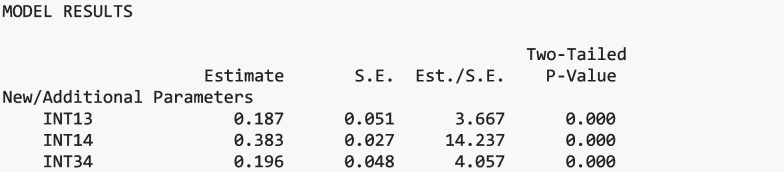


#### Within-class regressions

##### Slope coefficients

Additionally, each regression between the predictor and outcome was examined across class. The regressions in the four classes significantly differed from zero, *p* <.001. This implies that students' interest in science issues in each class significantly increases as their science performance increases.


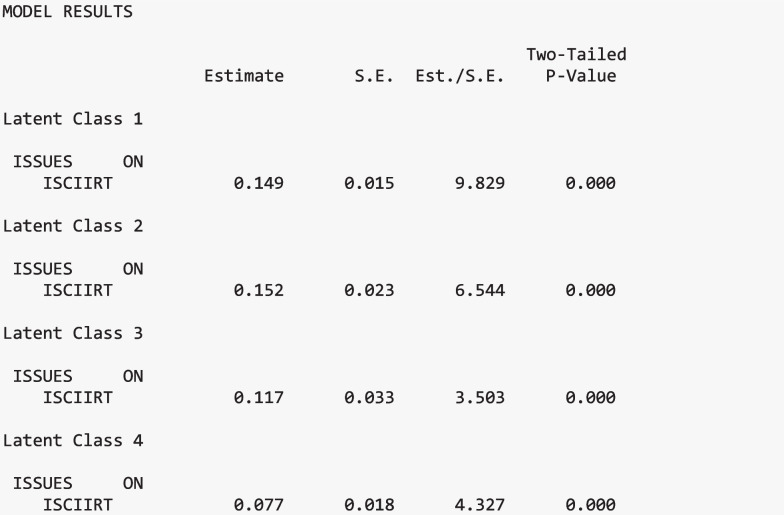


##### Intercept coefficients

When estimating class-specific means for the distal outcome (interest in science issues), the mean was fixed to zero for the *Ambivalent with Minimal Utility Value* class to allow for model identification. This class was used as the reference class. Thus, the factor's mean is set to zero, and others are compared to it. Compared to the *Ambivalent with Minimal Utility Value* class, students in the *Pro-Science with Elevated Utility* class demonstrated increased science ability (*M* = 0.246). In comparison, on average, students in the *Anti-Science with Minimal Utility* class performed lower on science (*M* = −0.137). The estimate for the *Ambivalent with Elevated Utility Value* class was not significant compared to the *Ambivalent with Minimal Utility Value* class.


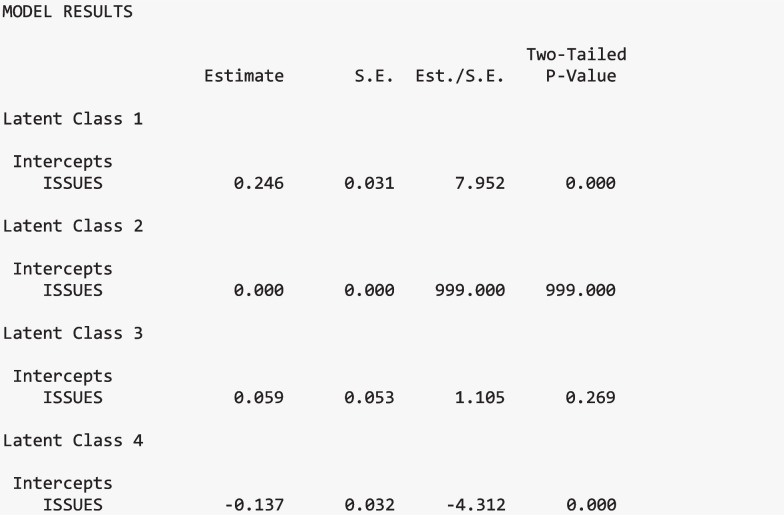


#### Covariates

The three covariates (URM, gender, and mother’s education) were included in the model and related to the distal outcome, thus serving as a control variable for other relations with the distal outcome. Table 6 presents the relations between the three covariates and the latent factor of interest in science issues. Only gender was a significant predictor of the distal latent outcome and interest in science issues, regardless of class. Specifically, on average, females report lower interest in science issues than males.

**Table 6:** Relations between the covariates and distal outcome

Covariate	Issues in science
Underrepresented minority	.043
Gender	−.160***
Mother’s education	.004

*p <.05, ***p <.001

In the Mplus output, these estimates appear separately for each class. However, because the model did not specify that these effects should vary across classes (as specified in the %OVERALL% section), the estimates are identical across all classes in the output. We had not hypothesized that this relation would vary across class, but it could vary if there is research interest in exploring class-specific relations with the covariate and distal outcomes.
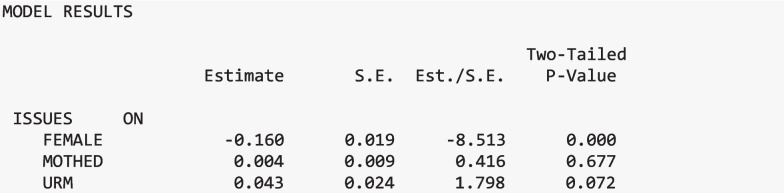


## Discussion

This paper provides a tutorial on moderation using a latent class variable, illustrated with an applied example. Specifically, we modeled the relationship between an exogenous (predictor or covariate) and endogenous (distal outcome) latent variable. The ML three-step method estimated the latent class variable simultaneously with the auxiliary variables. This enables the relationship between the predictor and distal outcome to vary across the latent classes while holding the measurement of the latent class variable constant. The following parameters were interpreted in the latent class moderation applied example:

1. The Wald chi-square test was used to test the equality of slopes across each latent class. A significant chi-square test provides evidence that moderation exists for the relationship between the predictor and outcome. Follow up with pairwise differences to assess where the interaction exists.
2. The Wald chi-square test to test the equality of the intercept, or means of the distal outcome, across each latent class. A significant chi-square test provides evidence of mean differences between groups on the distal outcome. Follow-up with pairwise differences to assess where the interaction exists.
3. Test the regression slopes and intercepts for each latent class. If significant, the slope or intercept in the class examined significantly differs from zero.

For the applied example, evidence of moderation was observed through a series of model testing steps. Table 2 outlines the modeling steps for applying this approach to other researchers. Moderation indicated a difference in relations between science achievement and interest in science issues across the latent classes of science attitudes. Since there are many class-specific parameters of the regression, there are multiple ways to test the equivalence of these parameters.

We demonstrated these comparisons using the LSAL example. Specifically, the example examined heterogeneity in 12th-grade students’ science attitudes and the relationship between their science achievement in 11th grade and their interest in science issues in 12th grade. We found that the relationship between science achievement and interest in science issues depends on the classes of 12th-grade science attitudes. Following these tests, pairwise comparisons of the slopes and intercepts were completed. Overall, students in all classes had positive slopes, indicating a positive relationship between science scores and their interest in science issues. Those in the *Anti-Science with Minimal Utility* class had statistically significant slope differences compared to those in the *Ambivalent with Elevated Utility* class and *Ambivalent with Minimal Utility* class. Finally, unsurprisingly, those in the *Pro-Science with Elevated Utility* class had a higher interest in science issues than those in the *Ambivalent with Elevated Utility* class and *Anti-Science with Minimal Utility* class.

This tutorial features a distal outcome modeled as a latent factor, but the code can be easily adapted for observed distal outcomes, in which case, it would not be necessary to fix one mean to zero. Alternative auxiliary variable methods, such as the BCH approach (Vermunt, 2010), can also be used to conduct latent class moderation. While this tutorial focuses on the ML three-step method for specification, interpretation, and visualization, much of the code structure is transferable to the BCH approach. Further, this example includes only one predictor and one distal outcome; the code can be extended to accommodate multiple covariates or distal outcomes. Finally, Asparouhov and Muthén (2014) and Vermunt (2010) demonstrated that the ML three-step approach is effective in preserving the latent class structure and producing unbiased estimates of covariate and distal outcome relationships under a range of conditions, provided that entropy or class separation is not too low. In the moderation context, Asparouhov and Muthén (2014) found that intercept and slope estimates were close to true parameter values, but not under varying conditions. Further research is warranted to assess the effectiveness of this approach in the context of moderation testing under a wider range of conditions.

### Moderation modeling extensions

This paper demonstrates how moderation can be modeled using a latent class variable as a moderator. Future pedagogical examples might extend this approach to incorporate additional auxiliary variables or interaction terms within class-specific models. In this context, the moderation would be interpreted as an interaction effect *within* each class that is allowed to *vary across* classes. Additionally, future applications could focus on different class-specific models. Exploring the utility of this modeling approach through critical frameworks (Gillborn et al., 2017) presents an important opportunity for future work (see Suzuki et al., 2021). In our applied example, we took a traditional approach by including demographic variables such as gender, ethnicity, and socioeconomic status, as single categorical covariates and regressing the outcome on these variables. This approach has been critiqued for its limitations, particularly for treating race and ethnicity as fixed, additive factors rather than socially constructed and structurally embedded categories. Such practices risk implying that race can be “controlled for,” potentially minimizing the structural significance of racialized experiences and leading to overly generic interpretations of group differences (James, 2001). While we included ethnicity as a control variable to account for students historically underrepresented in STEM fields, we acknowledge the limitations of this approach. One possible extension would be to use race or ethnicity as a grouping variable to examine whether the moderating relationships vary across racial or ethnic groups. We encourage researchers to move beyond traditional approaches and to take up more nuanced, critically informed quantitative strategies that better reflect the complexity of social identities and structures.

## Supplementary Information

Below is the link to the electronic supplementary material.

Supplementary file1 (PDF 490 KB)

Supplementary file2 (PDF 216 KB)

## Data Availability

The data and materials for all analyses are available on GitHub.

## Code availability

All code is available in the supplementary materials provided in this manuscript and on GitHub.
