# Supplementary material for: Moderation with a latent class variable: A tutorial and example
Source: Behav Res Methods. 2026 Apr 10;58(4):108. doi: 10.3758/s13428-025-02886-x (PMC13068770; doi:10.3758/s13428-025-02886-x)
Supplement: Supplementary file 1 — Supplementary file1 (PDF 490 KB) [file 13428_2025_2886_MOESM1_ESM.pdf]

Moderation with a latent class variable: A tutorial and example

Appendix A: R Code for the Manual ML Three-Step in M*plus*

Dina Arch

2025-04-21

R Code for the Manual ML Three-Step in M*plus*

This appendix walks through the R code to apply moderation with a latent class variable using the `MplusAutomation` package.

Packages

```
library(MplusAutomation)
library(tidyverse)
library(here)
library(glue)
library(gt)
library(cowplot)
library(kableExtra)
library(psych)
```

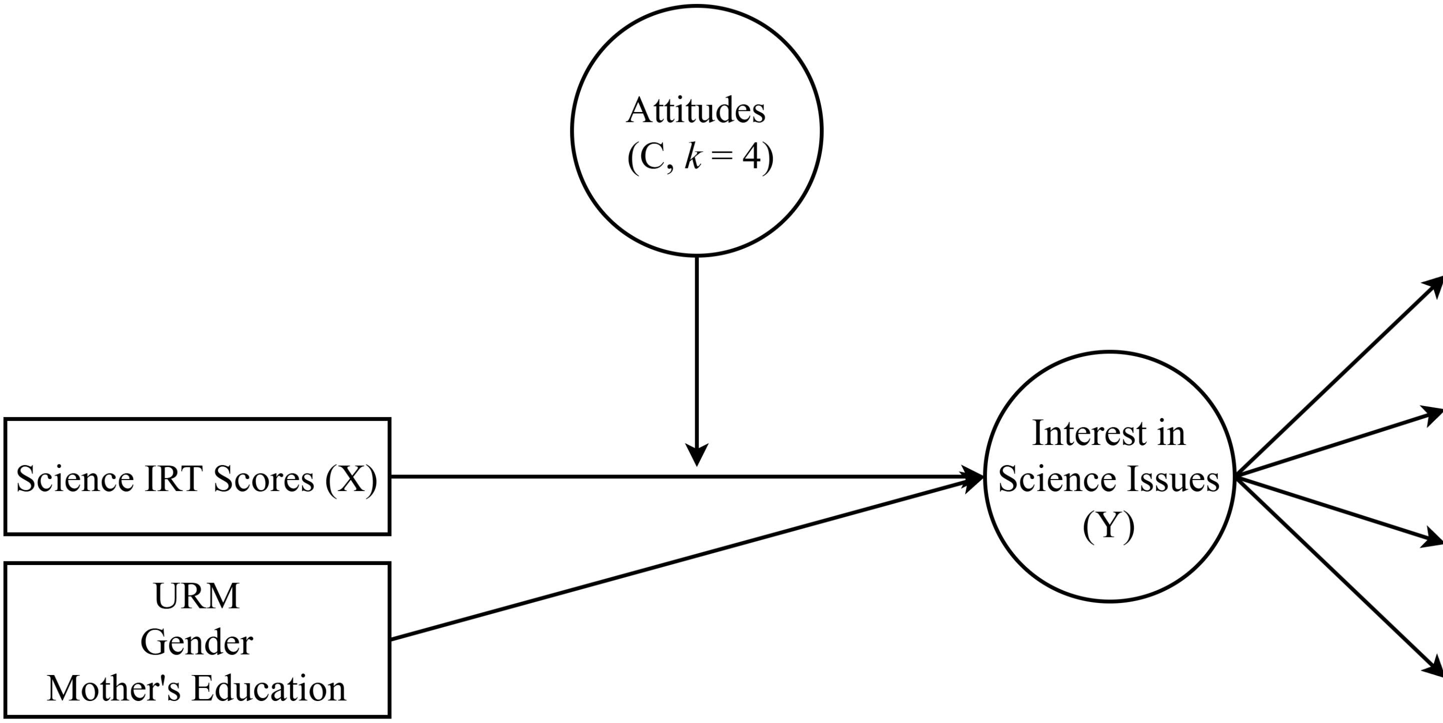

Table 1: Longitudinal Study of American Life

Name	Description
**LCA Indicator Variables**	
KA47A	I Enjoy Science
KA47H	Science is Useful in Everyday Problems
KA47I	Science Helps Logical Thinking
KA47K	Need Science for a Good Job
KA47L	Will Use Science Often as an Adult
**Predictor**	
ISCIIRT	Science IRT Score (11th Grade)
**Distal Outcome**	
KA9B	Space Exploration
KA9D	Scientific Discoveries
KA9G	New Technologies
KA9K	Energy Policy
**Covariates**	
URM	Under-represented Minority (0 = represented, 1 = under-represented)
FEMALE	Sex (0 = male, 1 = female)
MOTHED	Mother's Education (0 = less than high school, 1 = high school diploma, 2 = some college, 3 = 4-year co

Read in LSAL dataset

```r
data <- read_csv(here("data", "LSAL_data.csv"))
```

Descriptive Statistics

Descriptive Statistics using R:

Quick view of all the variables in the dataset (excluding `CASENUM`, `COHORT` and SCHOOLID‘):

```r
data %>%
  select(-CASENUM, -COHORT, -SCHOOLID) %>%
  describe()
```

Proportion of indicators using R:

```r
# Set up data to find proportions of binary indicators
ds <- data %>%
  pivot_longer(KA47A:KA47L, names_to = "Variable")

# Create table of variables and counts
tab <- table(ds$Variable, ds$value)

# Find proportions and round to 3 decimal places
prop <- prop.table(tab, margin = 1) %>%
  round(3)

# Combine everything to one table
dframe <- data.frame(Variables=rownames(tab), Proportion=prop[,2], Count=tab[,2])
#remove row names
row.names(dframe) <- NULL
```

```r
# Format table using `kable()`
dframe %>%
  kable(caption = "Descriptive Summary",booktabs = TRUE, escape = FALSE) %>%
  kableExtra::kable_styling(latex_options=c("HOLD_position"))
```

Table 2: Descriptive Summary

Variables	Proportion	Count
KA47A	0.534	1793
KA47H	0.450	1502
KA47I	0.548	1825
KA47K	0.341	1139
KA47L	0.403	1352

Descriptive Statistics using `MplusAutomation`:

```r
m.step0  <- mplusObject(
  TITLE = "LSAL Descriptive Statistics;",

  VARIABLE =
    "usevar = FEMALE MOTHED URM ISCIIRT KA9B KA9D KA9G KA9K
    KA47A KA47H KA47I KA47K KA47L;
    categorical = KA47A KA47H KA47I KA47K KA47L FEMALE MOTHED URM;",

#  DEFINE =  "ISCIIRT = ISCIIRT/10;
#    center ISCIIRT (GRANDMEAN);",

  ANALYSIS = "TYPE=basic;",

  OUTPUT = "sampstat",

  usevariables = colnames(data),
  rdata = data)

m.step0.fit <- mplusModeler(m.step0,
                            dataout = here("mplus", "LSAL_data.dat"),
                            modelout = here("mplus","basic.inp"),
                            check = TRUE, run = TRUE, hashfilename = FALSE)
```

View of descriptive statistics using `get_sampstat()`:

```r
# Using MplusAutomation
get_sampstat(m.step0.fit)

# Using base R
summary(data)
```

Or, view the `.out` file:

```
UNIVARIATE PROPORTIONS AND COUNTS FOR CATEGORICAL VARIABLES

    FEMALE
      Category 1    0.509        3026.000
      Category 2    0.491        2919.000
    MOTHED
      Category 1    0.147         854.000
      Category 2    0.580        3362.000
      Category 3    0.103         597.000
      Category 4    0.118         684.000
      Category 5    0.052         300.000
    URM
      Category 1    0.777        4313.000
      Category 2    0.223        1241.000
    KA47A
      Category 1    0.466        1564.000
      Category 2    0.534        1793.000
    KA47H
      Category 1    0.550        1837.000
      Category 2    0.450        1502.000
    KA47I
      Category 1    0.452        1507.000
      Category 2    0.548        1825.000
    KA47K
      Category 1    0.659        2200.000
      Category 2    0.341        1139.000
    KA47L
      Category 1    0.597        2003.000
      Category 2    0.403        1352.000
```

Enumeration

This code uses the `mplusObject` function in the `MplusAutomation` package and saves all model runs in the `mplus_enum` folder.

```
lca_6  <- lapply(1:6, function(k) {
  lca_enum  <- mplusObject(

    TITLE = glue("{k}-Class"),

    VARIABLE = glue(
      "categorical = KA47A KA47H KA47I KA47K KA47L;
     usevar = KA47A KA47H KA47I KA47K KA47L;
     classes = c({k});"),

    ANALYSIS =
```

```
      "estimator = mlr;
    type = mixture;
    processors = 12;
    starts = 500 100;",

    OUTPUT = "sampstat residual tech11 tech14;",

    usevariables = colnames(data),
    rdata = data)

  lca_enum_fit <- mplusModeler(lca_enum,
                             dataout=glue(here("enumeration", "LSAL_data.dat")),
                             modelout=glue(here("enumeration", "c{k}_lsal.inp")) ,
                             check=TRUE, run = TRUE, hashfilename = FALSE)
})
```

IMPORTANT: Before moving forward, make sure to examine each output document to ensure models were estimated normally. In this example, the last model (6-class models) did not produce reliable output and was excluded.

Table of Fit

First, extract data:

```
output_lsal <- readModels(here("enumeration"))

enum_extract <- LatexSummaryTable(
  output_lsal,
  keepCols = c(
    "Title",
    "Parameters",
    "LL",
    "BIC",
    "aBIC",
    "BLRT_PValue",
    "T11_VLMR_PValue",
    "Observations"
  ),
  sortBy = "Title"
) %>% slice_head(n=5) # Select first five models (Class 1 through 5)


allFit <- enum_extract %>%
  mutate(CAIC = -2* LL + Parameters * (log(Observations)+1)) %>%
  mutate(AWE = -2*LL+2*Parameters*(log(Observations)+1.5)) %>%
  mutate(SIC = -.5*BIC) %>%
  mutate(expSIC = exp(SIC - max(SIC))) %>%
  mutate(BF = exp(SIC - lead(SIC))) %>%
  mutate(cmPk = expSIC / sum(expSIC)) %>%
  dplyr::select(1:5, 9:10, 6:7, 13, 14) %>%
  arrange(Parameters)
```

Then, create table using `gt()` instead of `kable()`:

```
# Number of classes being evaluated
k <- 5

# Fit table
fit_table <- allFit %>%
  gt() %>%
  tab_header(title = "Table 1", subtitle = md("*Model Fit Summary Table*")) %>%
  cols_label(
    Title = "Classes",
    Parameters = md("Par"),
    LL = md("*LL*"),
    T11_VLMR_PValue = "VLMR",
    BLRT_PValue = "BLRT",
    BF = md("BF"),
    cmPk = md("*cmPk*")
  ) %>%
  tab_footnote(
    footnote = md(
      "*Note.* Par = Parameters; *LL* = model log likelihood;
BIC = Bayesian information criterion;
aBIC = sample size adjusted BIC; CAIC = consistent Akaike information criterion;
AWE = approximate weight of evidence criterion;
BLRT = bootstrapped likelihood ratio test p-value;
VLMR = Vuong-Lo-Mendell-Rubin adjusted likelihood ratio test p-value;
*cmPk* = approximate correct model probability."
    )
  ) %>%
  cols_align(align = "center") %>%
  opt_table_font(font = "Times New Roman") %>%
  opt_align_table_header(align = "left") %>%
  tab_options(
    heading.title.font.size = px(16),
    heading.subtitle.font.size = px(16),
    row_group.as_column = TRUE,
    stub.border.width = 0,
    heading.border.bottom.color = "black",
    column_labels.border.top.color = "black",
    column_labels.border.bottom.color = "black",
    table_body.border.bottom.color = "black",
    table.border.bottom.color = "white",
    table.border.top.color = "white",
    table.background.color = "white"
  ) %>%
  tab_style(
    style = list(
      cell_borders(
        sides = c("top", "bottom"),
        color = "white",
        weight = px(1)
      ),
      cell_text(align = "center"),
      cell_fill(color = "white", alpha = NULL)
```

```
  ),
  locations = cells_body(columns = everything(),
                         rows = everything())
) %>%
tab_options(column_labels.font.weight = "bold") %>%
fmt_number(c(3:7),
           decimals = 2) %>%
sub_missing(1:11,
            missing_text = "--") %>%
fmt(
  c(8:9, 11),
  fns = function(x)
    ifelse(x < .001, "< .001",
           scales::number(x, accuracy = .01))
) %>%
fmt(
  10,
  fns = function (x)
    ifelse(x > 100, ">100",
           scales::number(x, accuracy = .01))
) %>%
tab_style(
  style = list(
    cell_text(weight = "bold")
    ),
  locations = list(cells_body(
   columns = BIC,
   row = BIC == min(BIC[c(1:k)])
  ),
  cells_body(
   columns = aBIC,
   row = aBIC == min(aBIC[1:k])
  ),
  cells_body(
   columns = CAIC,
   row = CAIC == min(CAIC[1:k])
  ),
  cells_body(
   columns = AWE,
   row = AWE == min(AWE[1:k])
  ),
  cells_body(
   columns = cmPk,
   row =  cmPk == max(cmPk[1:k])
   ),
  cells_body(
   columns = BF,
   row =  BF > 10),
  cells_body(
   columns =  T11_VLMR_PValue,
   row =  ifelse(T11_VLMR_PValue < .05 & lead(T11_VLMR_PValue) > .05, T11_VLMR_PValue < .05, NA)),
  cells_body(
   columns =  BLRT_PValue,
```

Table 1

Model Fit Summary Table

Classes	Par	*LL*	BIC	aBIC	CAIC	AWE	BLRT	VLMR	BF
1-Class	5	-11,315.87	22,672.34	22,656.45	22,677.34	22,727.94	–	–	0.00
2-Class	11	-9,009.08	18,107.48	18,072.53	18,118.48	18,229.81	$< .001$	$< .001$	0.00
3-Class	17	-8,814.56	17,767.18	17,713.17	17,784.18	17,956.24	$< .001$	$< .001$	0.00
4-Class	23	-8,742.24	**17,671.26**	**17,598.17**	**17,694.26**	**17,927.04**	$< .001$	$< .001$	**>100**
5-Class	29	-8,734.82	17,705.15	17,613.01	17,734.15	18,027.66	$< .001$	0.01	–

Note. Par = Parameters; *LL* = model log likelihood; BIC = Bayesian information criterion; aBIC = sample size adjusted BIC; CAIC = consistent Akaike information criterion; AWE = approximate weight of evidence criterion; BLRT = bootstrapped likelihood ratio test p-value; VLMR = Vuong-Lo-Mendell-Rubin adjusted likelihood ratio test p-value; *cmPk* = approximate correct model probability.

```
     row =  ifelse(BLRT_PValue < .05 & lead(BLRT_PValue) > .05, BLRT_PValue < .05, NA))
  )
)

fit_table
```

Save table:

```
gtsave(fit_table, here("figures", "fit_table.png"))
```

Information Criteria Plot

```
allFit %>%
  dplyr::select(2:7) %>%
  rowid_to_column() %>%
  pivot_longer(`BIC`:`AWE`,
               names_to = "Index",
               values_to = "ic_value") %>%
  mutate(Index = factor(Index,
                        levels = c ("AWE", "CAIC", "BIC", "aBIC"))) %>%
  ggplot(aes(
    x = rowid,
    y = ic_value,
    color = Index,
    shape = Index,
    group = Index,
```

```
  lty = Index
)) +
geom_point(size = 2.0) + geom_line(size = .8) +
scale_x_continuous(breaks = 1:nrow(allFit)) +
scale_colour_grey(end = .5) +
theme_cowplot() +
labs(x = "Number of Classes", y = "Information Criteria Value", title = "Information Criteria") +
theme(
  text = element_text(family = "serif", size = 12),
  legend.text = element_text(family="serif", size=12),
  legend.key.width = unit(3, "line"),
  legend.title = element_blank(),
  legend.position = "top"
)
```

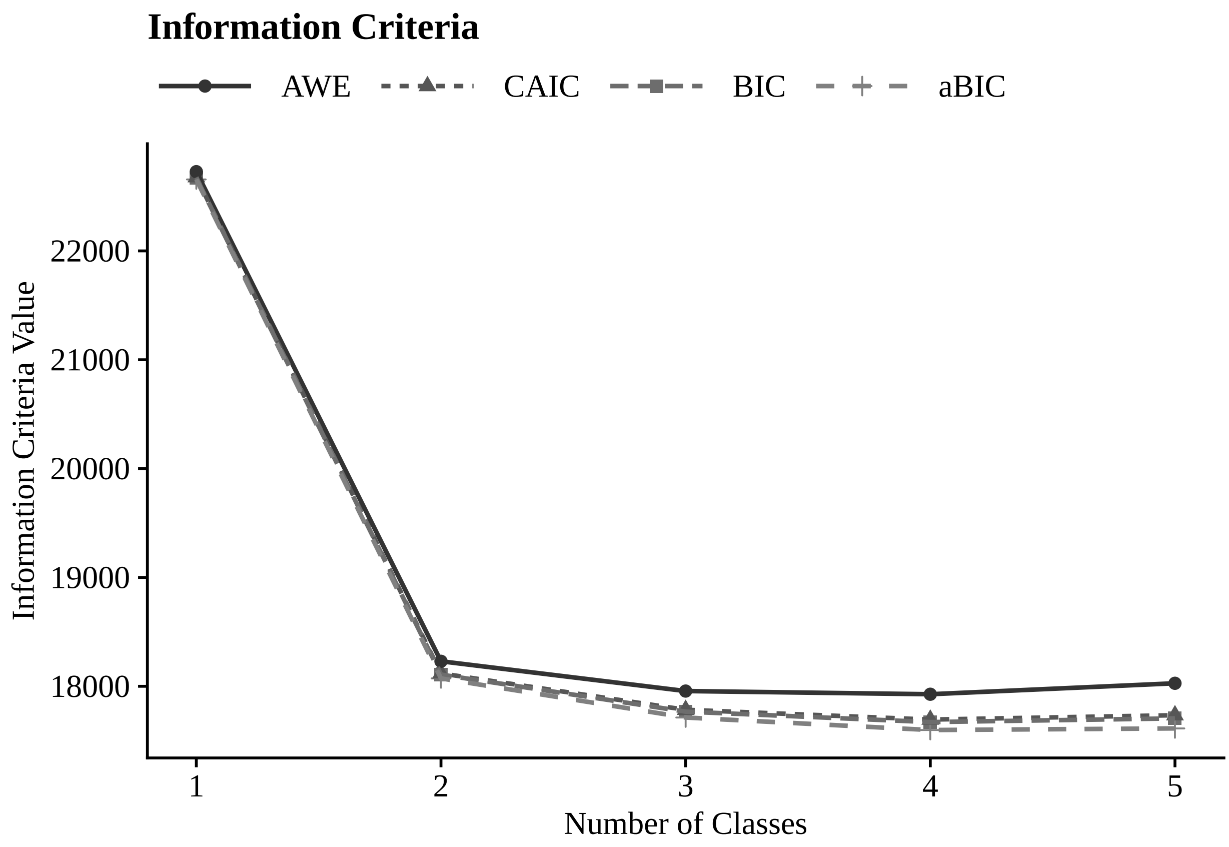

Save figure:

```
ggsave(here("figures", "info_criteria.png"), dpi=300, height=5, width=7, units="in")
```

Compare Class Solutions

Compare probability plots for $K = 1:5$ class solutions

```
model_results <- data.frame()

for (i in 1:length(output_lsal)) {
  temp <- output_lsal[[i]]$parameters$probability.scale %>%
    mutate(model = paste0(i, "-Class Model"))

  model_results <- rbind(model_results, temp)
}

compare_plot <-
  model_results %>%
  filter(category == 2) %>%
  dplyr::select(est, model, LatentClass, param) %>%
  filter(model != "6-Class Model") #Remove from plot

compare_plot$param <- fct_inorder(compare_plot$param)

ggplot(
  compare_plot,
  aes(
    x = param,
    y = est,
    color = LatentClass,
    shape = LatentClass,
    group = LatentClass,
    lty = LatentClass
  )
) +
  geom_point() +
  geom_line() +
  scale_colour_viridis_d() +
  facet_wrap(~ model, ncol = 2) +
  labs(title = "Math Attitude Items", x = " ", y = "Probability") +
  theme_minimal() +
  theme(panel.grid.major.y = element_blank(),
        axis.text.x = element_text(angle = -45, hjust = -.1))
```

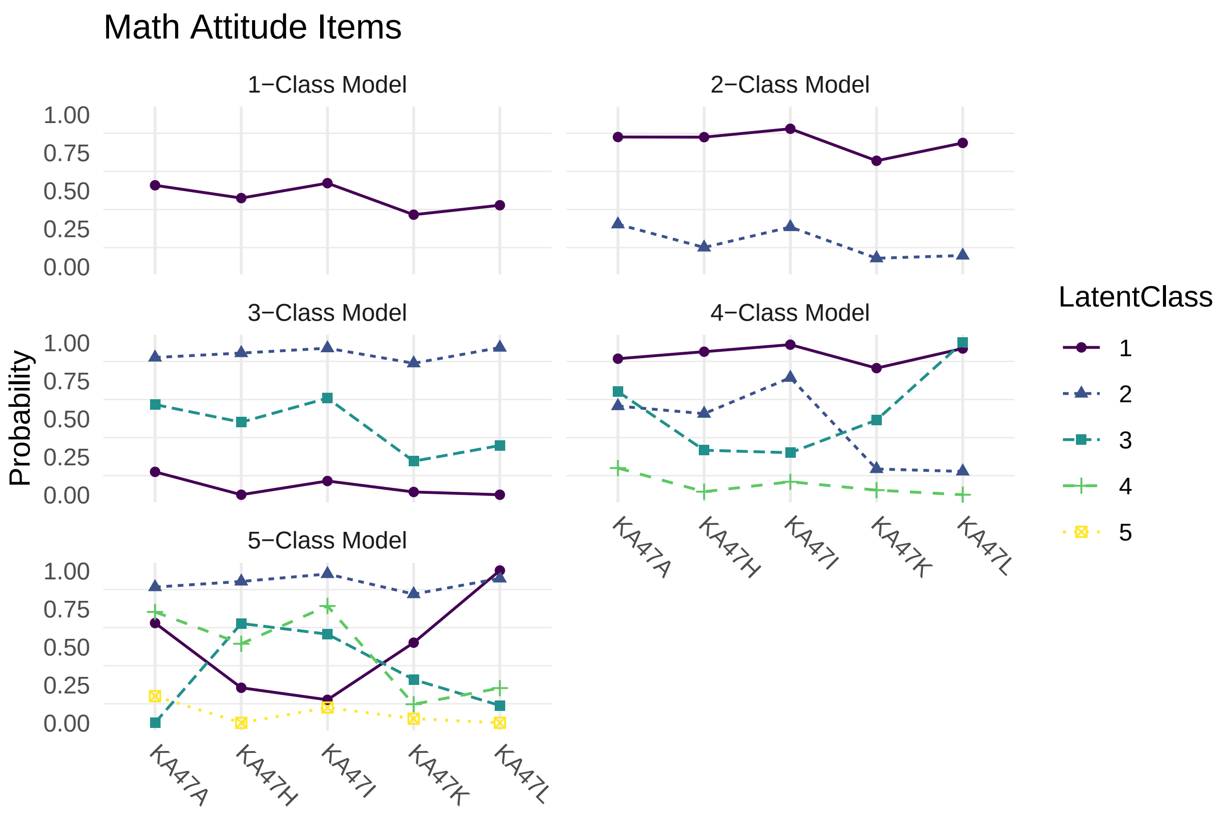

Save figure:

```
ggsave(here("figures", "compare_kclass_plot.png"), dpi=300, height=5, width=7, units="in")
```

4-Class Probability Plot

Use the `plot_lca` function provided in the folder to plot the item probability plot. This function requires one argument: - `model_name`: The name of the Mplus `readModels` object (e.g., `output_lsal$c4_lsal.out`)

```
source("plot_lca.txt")

plot_lca(model_name = output_lsal$c4_lsal.out)
```

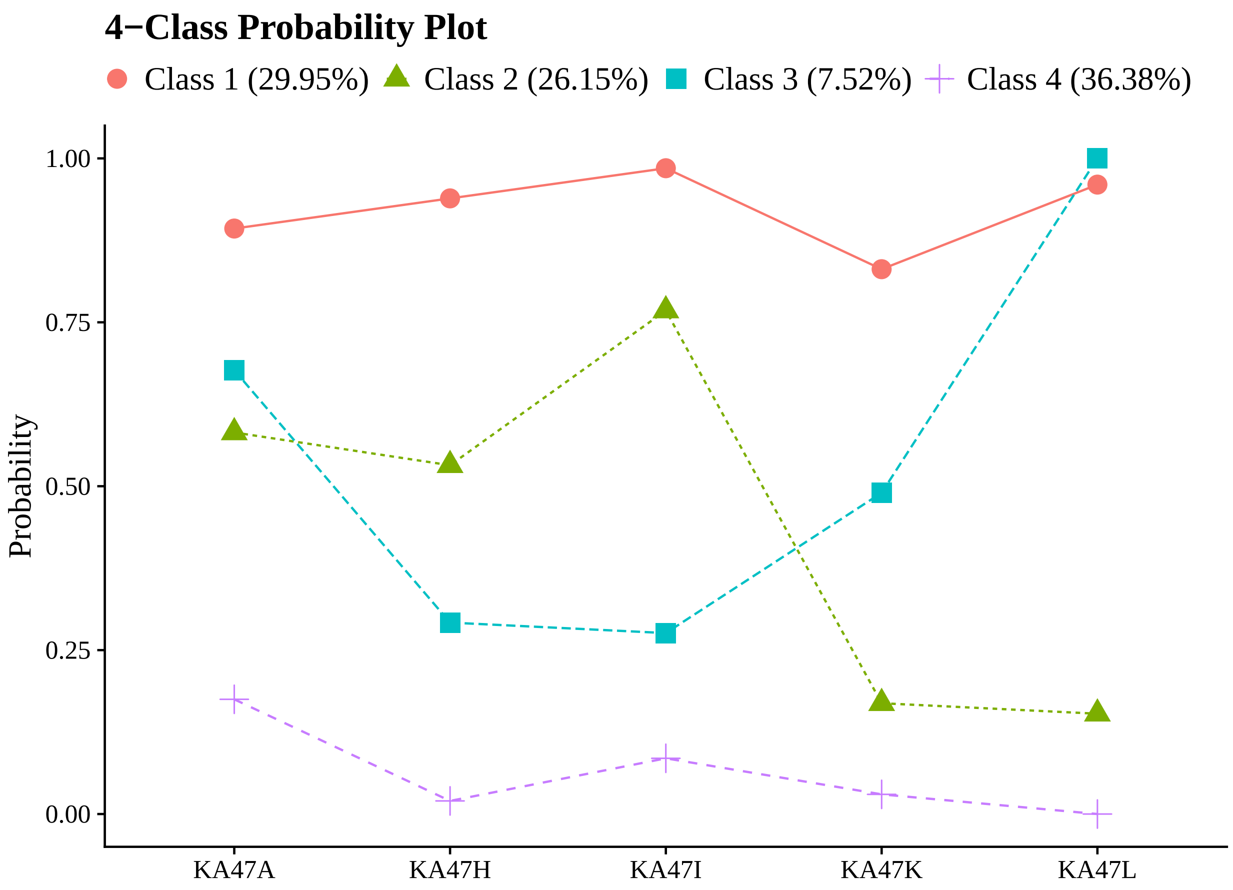

Save figure:

```
ggsave(here("figures", "probability_plot.png"), dpi = "retina", bg = "white", height=5, width=7, units=
```

Latent class variable as moderator

Step 1 - Class Enumeration w/ Auxiliary Specification

```
step1  <- mplusObject(
  TITLE = "Step 1 - Class Enumeration w/ Auxiliary Specification",
  VARIABLE = "categorical = KA47A KA47H KA47I KA47K KA47L;
  usevar =  KA47A KA47H KA47I KA47K KA47L;
```

```
  classes = c(4);
  AUXILIARY = FEMALE MOTHED ISCIIRT KA9B KA9D KA9G KA9K URM;",

  ANALYSIS =
   "estimator = mlr;
    type = mixture;
    processors = 12;
    OPTSEED = 573096;",

  SAVEDATA =
   "File=savedata.dat;
    Save=cprob;
    format=free;",

  OUTPUT = "sampstat residual tech11 tech14",

  usevariables = colnames(data),
  rdata = data)

step1_fit <- mplusModeler(step1,
                            dataout=here("three_step", "new.dat"),
                            modelout=here("three_step", "one.inp") ,
                            check=TRUE, run = TRUE, hashfilename = FALSE)
```

Note: Ensure that the classes did not shift during this step (i.e., Class 1 in the enumeration run is now Class 4). Evaluate output and compare the class counts and proportions for the latent classes. Using the OPTSEED function ensures replication of the best loglikelihood value run.

After selecting the latent class model, add class labels to item probability plot using the `plot_lca_labels` function. This function requires three arguments:

- `model_name`: The Mplus `readModels` object (e.g., `output_lsal$c4_lsal.out`)
- `item_labels`: The item labels for x-axis (e.g.,c("Enjoy","Useful","Logical","Job","Adult"))
- `class_labels`: The class labels (e.g., c("Pro-Science w/ Elevated Utility Value", "Ambivalent w/ Minimal Utility Value", "Ambivalent w/ Elevated Utility Value", "Anti-Science w/ Minimal Utility Value"))

Note: Use `\n` to add a return if the label is lengthy.

```
source("plot_lca_labels.txt")

# Read in output from step 1.
output_lsal <- readModels(here("three_step","one.out"))

# Plot Title
title <- "LCA Probability Plot - LSAL"

#Identify item and class labels (Make sure they are in the order presented in the plot above)
item_labels <-  c(
  "I Enjoy \nScience",
```

```r
  "Science is Useful \nin Everyday Problems",
  "Science Helps \nLogical Thinking",
  "Need Science for \na Good Job",
  "Will Use Science \nOften as an Adult"
)

class_labels <- c(
  "Pro-Science w/ \nElevated Utility Value",
  "Ambivalent w/ \nMinimal Utility Value",
  "Ambivalent w/ \nElevated Utility Value",
  "Anti-Science w/ \nMinimal Utility Value"
)

# Plot LCA plot
plot_lca_labels(model_name = output_lsal, item_labels, class_labels, title)
```

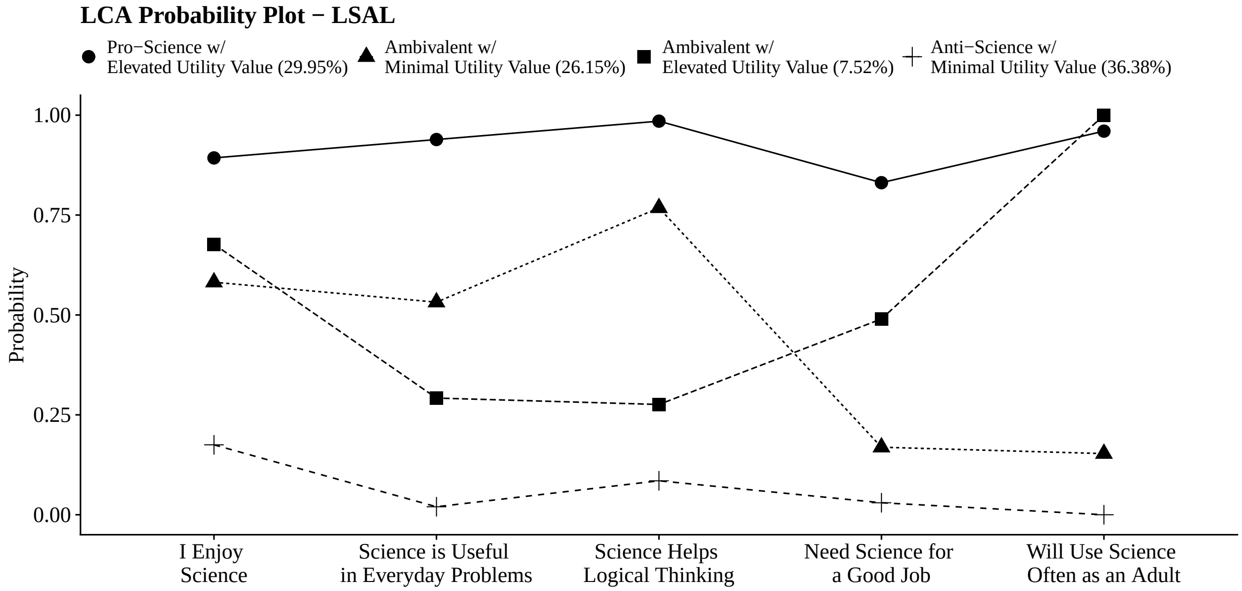


```r
# Save
ggsave(here("figures", "final_probability_plot.png"), dpi = "retina", bg = "white", height=7, width=10,
```

Step 2 - Determine Measurement Error

Extract logits for the classification probabilities for the most likely latent class:

```r
logit_cprobs <- as.data.frame(output_lsal[["class_counts"]]
                                        [["logitProbs.mostLikely"]])
```

Extract saved dataset from step one:

```r
savedata <- as.data.frame(output_lsal[["savedata"]])
```

Rename the column in savedata named "C" and change to "N":

```r
colnames(savedata)[colnames(savedata)=="C"] <- "N"
```

Step 3 - Add Auxiliary Variables

To test for moderation, an overall test of equivalence of the regression of science issues on science ability across the latent classes was conducted using the omnibus Wald test. This is done in `MplusAutomation` using the `MODELTEST` command shown below. Mplus can run only one Wald test at a time. After evaluating the first Wald test (slopes), re-run step three for the second Wald test (intercepts). Pairwise comparisons can be tested simultaneously, but should be evaluated after significant Wald tests.

```r
step3mod  <- mplusObject(
  TITLE = "Step 3 - LSAL Moderation",

  VARIABLE =
  "USEVAR = FEMALE MOTHED ISCIIRT URM KA9B KA9D KA9G KA9K N;
  classes = c(4);
  nominal = N;",

  ANALYSIS =
 "estimator = mlr;
  type = mixture;
  starts = 0;
  iterations = 1000;",

  DEFINE =
  "ISCIIRT = ISCIIRT/10;
   Center ISCIIRT (GRANDMEAN);",

  MODEL =
  glue("
!Covariates: URM FEMALE MOTHED ISCIIRT
!Distal: ISSUES

  %OVERALL%
  ISSUES by KA9B KA9D KA9G KA9K;
  ISSUES on FEMALE MOTHED URM;
  ISSUES on ISCIIRT;

         %C#1%
[N#1@{logit_cprobs[1,1]}];
[N#2@{logit_cprobs[1,2]}];
[N#3@{logit_cprobs[1,3]}];
      [ISSUES] (B01);        ! conditional distal mean
```

```
      ISSUES;                 ! conditional distal variance (freely estimated)
      ISSUES on ISCIIRT(B11);! conditional slope (class 1)

        %C#2%
[N#1@{logit_cprobs[2,1]}];
[N#2@{logit_cprobs[2,2]}];
[N#3@{logit_cprobs[2,3]}];
      [ISSUES@0] (B02);
      ISSUES;
      ISSUES on ISCIIRT(B12);

        %C#3%
[N#1@{logit_cprobs[3,1]}];
[N#2@{logit_cprobs[3,2]}];
[N#3@{logit_cprobs[3,3]}];
      [ISSUES] (B03);
      ISSUES;
      ISSUES on ISCIIRT(B13);

        %C#4%
[N#1@{logit_cprobs[4,1]}];
[N#2@{logit_cprobs[4,2]}];
[N#3@{logit_cprobs[4,3]}];
      [ISSUES] (B04);
      ISSUES;
      ISSUES on ISCIIRT(B14);"),

MODELTEST = "
! can run only a single Omnibus test per model
    ! Omnibus test 1 (Slope)
       B11=B12;
       B12=B13;
       B13=B14;
    ! Omnibus test 2 (Intercept)
       !B01=B03;
       !B03=B04;",

  MODELCONSTRAINT =
   "NEW (slope12, slope13, slope14, slope23, slope24, slope34,
           int13, int14, int34);

      slope12=B11-B12; ! Test slope differences
      slope13=B11-B13;
      slope14=B11-B14;
      slope23=B12-B13;
      slope24=B12-B14;
      slope34=B13-B14;

      int13=B01-B03; ! Test intercept differences
      int14=B01-B04;
      int34=B03-B04;",

  usevariables = colnames(savedata),
```

```
  rdata = savedata)

step3mod_fit <- mplusModeler(step3mod,
               dataout=here("three_step", "new.dat"),
               modelout=here("three_step", "three.inp"),
               check=TRUE, run = TRUE, hashfilename = FALSE)
```

Wald Test of Paramter Constraints (Slope)

Wald Test (*df*)	*p*-value
11.003 (3)	0.012*

**Latent Class**	**Label**
1	Pro-Science with Elevated Utility Value
2	Ambivalent with Minimal Utility Value
3	Ambivalent with Elevated Utility Value
4	Anti-Science with Minimal Utility Value

```r
modelParams <- readModels(here("three_step", "three.out"))

# Extract information as data frame
wald <- as.data.frame(modelParams[["summaries"]]) %>%
  dplyr::select(WaldChiSq_Value:WaldChiSq_PValue) %>%
  mutate(WaldChiSq_DF = paste0("(", WaldChiSq_DF, ")")) %>%
  unite(wald_test, WaldChiSq_Value, WaldChiSq_DF, sep = " ") %>%
  rename(pval = WaldChiSq_PValue) %>%
  mutate(pval = ifelse(pval<0.001, paste0(".001*"),
                       ifelse(pval<0.05, paste0(scales::number(pval, accuracy = .001), "*"),
                              scales::number(pval, accuracy = .001))))

# Create table

wald %>%
  gt() %>%
    tab_header(
    title = "Wald Test of Paramter Constraints (Slope)") %>%
    cols_label(
      wald_test = md("Wald Test (*df*)"),
      pval = md("*p*-value")) %>%
  cols_align(align = "center") %>%
  opt_align_table_header(align = "left") %>%
  gt::tab_options(table.font.names = "serif")
```

Wald Test Table

Table of Slope and Intercept Values Across Classes See Table 5 in manuscript for an organized table of the slope and intercept values across science attitude classes.

```r
modelParams <- readModels(here("three_step", "three.out"))

# Extract information as data frame
values <- as.data.frame(modelParams[["parameters"]][["unstandardized"]]) %>%
  filter(param %in% c("ISSUES", "ISCIIRT"),
         paramHeader != "Residual.Variances") %>%
  mutate(param = str_replace(param, pattern = "ISCIIRT", replacement = "Slope"),
         param = str_replace(param, pattern = "ISSUES", replacement = "Intercept")) %>%
  mutate(LatentClass = sub("^","Class ", LatentClass)) %>%
  dplyr::select(!paramHeader) %>%
  mutate(se = paste0("(", format(round(se,2), nsmall =2), ")")) %>%
    unite(estimate, est, se, sep = " ") %>%
  select(!est_se) %>%
  mutate(pval = ifelse(pval<0.001, paste0("<.001*"),
                       ifelse(pval<0.05, paste0(scales::number(pval, accuracy = .001), "*"),
                              scales::number(pval, accuracy = .001))))


# Create table

values %>%
  gt(groupname_col = "LatentClass", rowname_col = "param") %>%
  tab_header(
    title = "Slope and Intercept Values Across Science Attitudes Classes") %>%
  cols_label(
    estimate = md("Estimate (*se*)"),
    pval = md("*p*-value")) %>%
  sub_values(values = "999.000", replacement = "-") %>%
  sub_missing(1:3,
              missing_text = "") %>%
  cols_align(align = "center") %>%
  opt_align_table_header(align = "left") %>%
  gt::tab_options(table.font.names = "serif")
```

```r
modelParams <- readModels(here("three_step", "three.out"))

# Extract information as data frame
diff1 <- as.data.frame(modelParams[["parameters"]][["unstandardized"]]) %>%
  filter(grepl("INT", param)) %>%
  dplyr::select(param:pval) %>%
  mutate(se = paste0("(", format(round(se,2), nsmall =2), ")")) %>%
  unite(estimate, est, se, sep = " ") %>%
  mutate(param = str_remove(param, "INT"),
         param = as.numeric(param)) %>%
  separate(param, into = paste0("Group", 1:2), sep = 1) %>%
  mutate(class = paste0("Class ", Group1, " vs ", Group2)) %>%
  select(class, estimate, pval) %>%
  mutate(pval = ifelse(pval<0.001, paste0("<.001*"),
```

Slope and Intercept Values Across Science Attitudes Classes

	Estimate (*se*)	*p*-value
Class 1		
Slope	0.149 (0.01)	<.001*
Intercept	0.246 (0.03)	<.001*
Class 2		
Slope	0.152 (0.02)	<.001*
Intercept	0 (0.00)	-
Class 3		
Slope	0.117 (0.03)	<.001*
Intercept	0.059 (0.05)	0.269
Class 4		
Slope	0.077 (0.02)	<.001*
Intercept	-0.137 (0.03)	<.001*

Distal Outcome Differences

Class	Mean (*se*)	*p*-value
Class 1 vs 3	0.187 (0.05)	<.001*
Class 1 vs 4	0.383 (0.03)	<.001*
Class 3 vs 4	0.196 (0.05)	<.001*

```
                       ifelse(pval<0.05, paste0(scales::number(pval, accuracy = .001), "*"),
                              scales::number(pval, accuracy = .001))))


# Create table

diff1 %>%
  gt() %>%
    tab_header(
    title = "Distal Outcome Differences") %>%
    cols_label(
      class = "Class",
      estimate = md("Mean (*se*)"),
      pval = md("*p*-value")) %>%
    sub_missing(1:3,
              missing_text = "") %>%
  cols_align(align = "center") %>%
  opt_align_table_header(align = "left") %>%
  gt::tab_options(table.font.names = "serif")
```

Table of Distal Outcome Differences

Slope Differences

Class	Mean (*se*)	*p*-value
Class 1 vs 2	-0.003 (0.03)	0.924
Class 1 vs 3	0.032 (0.04)	0.388
Class 1 vs 4	0.072 (0.02)	0.001*
Class 2 vs 3	0.035 (0.04)	0.419
Class 2 vs 4	0.075 (0.03)	0.019*
Class 3 vs 4	0.04 (0.04)	0.285

```r
modelParams <- readModels(here("three_step", "three.out"))

# Extract information as data frame
diff2 <- as.data.frame(modelParams[["parameters"]][["unstandardized"]]) %>%
  filter(grepl("SLOPE", param)) %>%
  dplyr::select(param:pval) %>%
  mutate(se = paste0("(", format(round(se,2), nsmall =2), ")")) %>%
  unite(estimate, est, se, sep = " ") %>%
  mutate(param = str_remove(param, "SLOPE"),
         param = as.numeric(param)) %>%
  separate(param, into = paste0("Group", 1:2), sep = 1) %>%
  mutate(class = paste0("Class ", Group1, " vs ", Group2)) %>%
  select(class, estimate, pval) %>%
  mutate(pval = ifelse(pval<0.001, paste0("<.001*"),
                       ifelse(pval<0.05, paste0(scales::number(pval, accuracy = .001), "*"),
                              scales::number(pval, accuracy = .001))))


# Create table

diff2 %>%
  gt() %>%
    tab_header(
    title = "Slope Differences") %>%
    cols_label(
      class = "Class",
      estimate = md("Mean (*se*)"),
      pval = md("*p*-value")) %>%
    sub_missing(1:3,
              missing_text = "") %>%
  cols_align(align = "center") %>%
  opt_align_table_header(align = "left") %>%
  gt::tab_options(table.font.names = "serif")
```

Table of Slope Differences

Relations Between the Covariates and Distal Outcome

	Estimate (*se*)	*p*-value
Gender	-0.16 (0.02)	<.001*
Mother's Education	0.004 (0.01)	0.677
Underrepresented Minority	0.043 (0.02)	0.072

```
modelParams <- readModels(here("three_step", "three.out"))

# Extract information as data frame
cov <- as.data.frame(modelParams[["parameters"]][["unstandardized"]]) %>%
  filter(param %in% c("FEMALE", "MOTHED", "URM")) %>%
  mutate(param = str_replace(param, "FEMALE", "Gender"),
         param = str_replace(param, "MOTHED", "Mother's Education"),
         param = str_replace(param, "URM", "Underrepresented Minority"))%>%
  mutate(LatentClass = sub("^","Class ", LatentClass)) %>%
  dplyr::select(!paramHeader) %>%
  mutate(se = paste0("(", format(round(se,2), nsmall =2), ")")) %>%
    unite(estimate, est, se, sep = " ") %>%
  select(param, estimate, pval) %>%
  distinct(param, .keep_all=TRUE)  %>%
  mutate(pval = ifelse(pval<0.001, paste0("<.001*"),
                      ifelse(pval<0.05, paste0(scales::number(pval, accuracy = .001), "*"),
                             scales::number(pval, accuracy = .001))))


# Create table

cov %>%
  gt(groupname_col = "LatentClass", rowname_col = "param") %>%
  tab_header(
    title = "Relations Between the Covariates and Distal Outcome") %>%
  cols_label(
    estimate = md("Estimate (*se*)"),
    pval = md("*p*-value")) %>%
  sub_missing(1:3,
              missing_text = "") %>%
  sub_values(values = c(999.000), replacement = "-") %>%
  cols_align(align = "center") %>%
  opt_align_table_header(align = "left") %>%
  gt::tab_options(table.font.names = "serif")
```

Table of Covariates

```
modelParams <- readModels(here("three_step", "three.out"))
```

```r
# Extract class size
c_size <- as.data.frame(modelParams[["class_counts"]][["modelEstimated"]][["proportion"]]) %>%
  rename("cs" = 1) %>%
  mutate(cs = round(cs*100, 2))

# Keep this code if you want a generic label for the classes
#c_size_val <- paste0("C", 1:nrow(c_size), glue(" ({c_size[1:nrow(c_size),]}%)"))

# Otherwise use this:
c_size_val <- paste0(class_labels, glue(" ({c_size[1:nrow(c_size),]}%)"))


# Extract information as data frame
estimates <- as.data.frame(modelParams[["parameters"]][["unstandardized"]]) %>%
  filter(paramHeader == "Intercepts") %>%
  dplyr::select(param, est, se) %>%
  filter(param == "ISSUES") %>% # Distal Outcome Name
  mutate(across(c(est, se), as.numeric)) %>%
  mutate(LatentClass = c_size_val)

# Add labels (NOTE: You must change the labels to match the significance testing!!)
#value_labels <- paste0(estimates$est, c("a"," bc"," abd"," cd"))

estimates$LatentClass <- fct_inorder(estimates$LatentClass)

# Plot bar graphs
estimates %>%
  ggplot(aes(x=LatentClass, y = est, fill = LatentClass)) +
  geom_col(position = "dodge", stat = "identity", color = "black") +
  geom_errorbar(aes(ymin=est-se, ymax=est+se),
                size=.3,    # Thinner lines
                width=.2,
                position=position_dodge(.9)) +
#  scale_fill_grey(start = .4, end = .7) + # Remove for colorful bars
  labs(y="Interest in Science Issues", x="") +
  theme_cowplot() +
  theme(text = element_text(family = "serif", size = 15),
        axis.text.x = element_text(size=15),
        legend.position="none")
```

Plot Distal Outcome

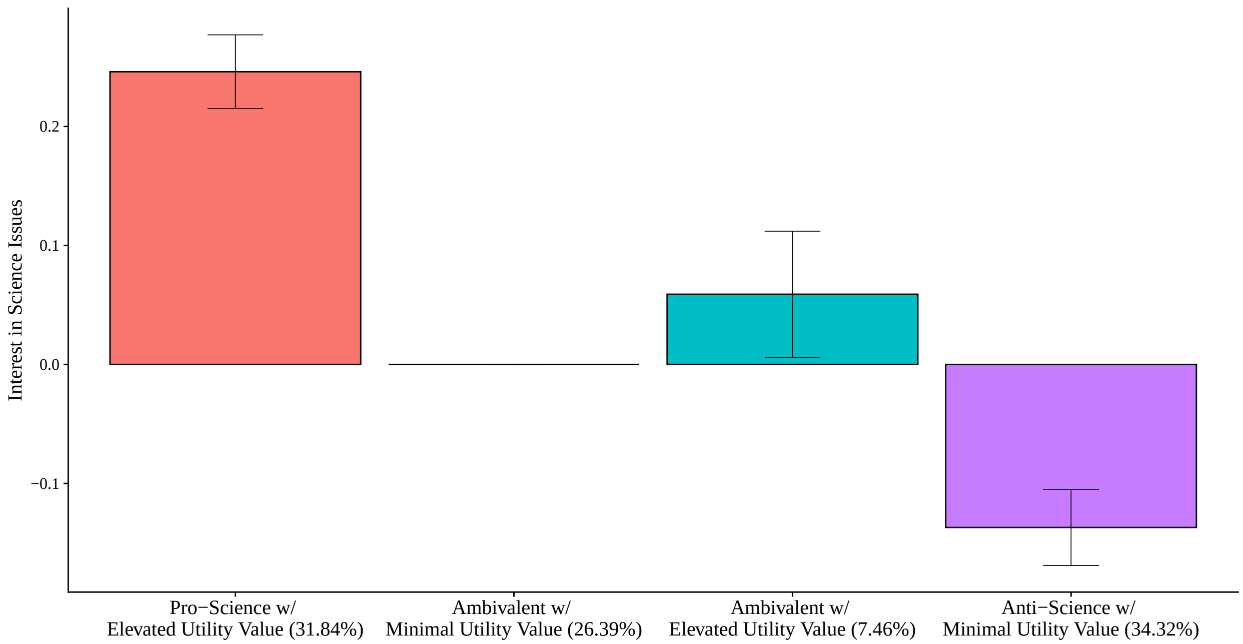


```
# Save plot
ggsave(here("figures","distal_plot.jpg"), dpi = "retina", bg = "white",  width=13, height = 7, units="i
```

```
modelParams <- readModels(here("three_step", "three.out"))

# Minimum and Maximum Values
desc <- as.data.frame(modelParams$sampstat$univariate.sample.statistics) %>%
  rownames_to_column("Variables")

# Select min amd max values of covariate
xmin <- desc %>%
  filter(Variables == "ISCIIRT") %>%
  dplyr::select(Minimum) %>%
  as.numeric()
xmax <- desc %>%
  filter(Variables == "ISCIIRT") %>%
  dplyr::select(Maximum) %>%
  as.numeric()

# Add slope and intercept, Min and Max values
line <- as.data.frame(modelParams$parameters$unstandardized) %>%
  filter(str_detect(paramHeader, 'ON|Inter'),
         str_detect(param, 'ISCIIRT|ISSUES')) %>% # ISCIIRT is X and ISSUES is Y
  unite("param", paramHeader:param, remove = TRUE) %>%
  mutate(param = replace(param,agrep(".ON",param),"slope"),
         param = replace(param,agrep("Inter", param), "intercept"),
```

```
       LatentClass = factor(LatentClass, labels = c_size_val)) %>% # Uses previous `c_size_val` objec
  dplyr::select(param, est, LatentClass) %>%
  pivot_wider(names_from = param, values_from = est) %>%
  add_column(x_max = xmax,
       x_min = xmin)


# Add column with y values
plot_data <- line %>%
  mutate(y_min = (slope*xmin) + intercept,
       y_max = (slope*xmax) + intercept) %>%
  dplyr::select(-slope, -intercept) %>%
  pivot_longer(-LatentClass,
             names_to = c("xvalues", "yvalues"),
             names_sep="_" ) %>%
  pivot_wider(names_from = xvalues, values_from = value) %>%
  dplyr::select(-yvalues)

# un-center and un-scale so values on x-axis are on the original scale
sampstat <- readModels(here("mplus", "basic.out"))
desc <- as.data.frame(sampstat$sampstat$univariate.sample.statistics) %>%
  rownames_to_column("Variables")
mean <- desc %>%
  filter(Variables == "ISCIIRT") %>%
  select(Mean) %>%
  as.numeric()
  uncentered_data <- plot_data %>% mutate(x = x*10 + mean)


# Plot
uncentered_data %>%
  ggplot(aes(
    x = x,
    y = y,
    color = LatentClass,
    group = LatentClass,
    lty = LatentClass,
    shape = LatentClass
  )) +
  geom_point(size = 4) +
  geom_line(aes(group = LatentClass), size = 1) +
  labs(x = "Interest in Science Issues",
      y = "Math Score") +
  scale_colour_grey(start = 0, end = 0) +
  scale_x_continuous(n.breaks = 10, limits = c(20,100)) +
  theme_cowplot() +
  theme(
    text = element_text(family = "serif", size = 15),
    axis.text.x = element_text(size = 15),
    legend.position = "top",
    legend.title = element_blank()
  )
```

Plot Slopes

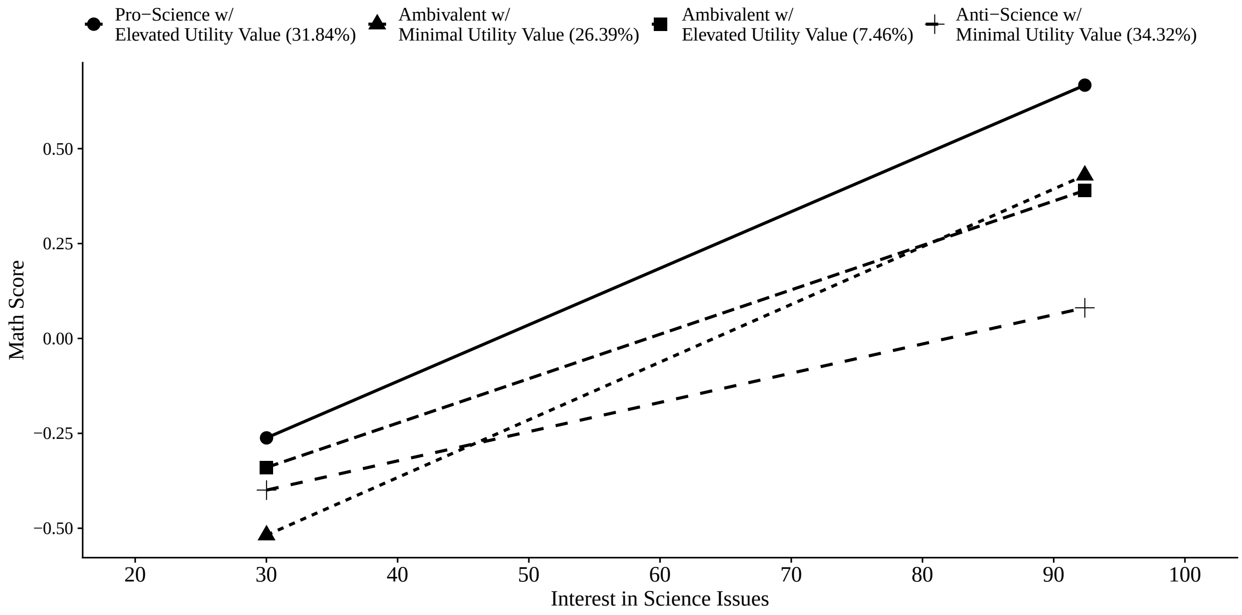


```
# Save
ggsave(here("figures","slope_plot.jpg"), dpi = "retina", bg = "white", width=11, height = 7, units="in")
```
