# Supplementary material for: Moderation with a latent class variable: A tutorial and example
Source: Behav Res Methods. 2026 Apr 10;58(4):108. doi: 10.3758/s13428-025-02886-x (PMC13068770; doi:10.3758/s13428-025-02886-x)
Supplement: Supplementary file 2 — Supplementary file2 (PDF 216 KB) [file 13428_2025_2886_MOESM2_ESM.pdf]

Appendix B: Annotated Output

Annotated Output of the Manual ML Three-Step in M*plus*

This Appendix walks through the output of three different Mplus runs using the Longitudinal Study of American Life (LSAL) example: descriptive statistics, class enumeration, and moderation model. Only relevant output is shown that corresponds to what is discussed in the paper. Below is a table with variable names and their description. Note: Comments in brown are notes and not part of the syntax. Notes can be included in Mplus using exclamation points.

Table 1: Longitudinal Study of American Life

Name	Description
**LCA Indicator Variables**	
KA47A	I Enjoy Science
KA47H	Science is Useful in Everyday Problems
KA47I	Science Helps Logical Thinking
KA47K	Need Science for a Good Job
KA47L	Will Use Science Often as an Adult
**Predictor**	
ISCIIRT	Science IRT Score (11th Grade)
**Distal Outcome**	
KA9B	Space Exploration
KA9D	Science Issues
KA9G	New Technologies
KA9K	Energy Policy Issues
**Covariates**	
URM	Under-represented Minority (0 = represented, 1 = under-represented)
FEMALE	Sex (0 = male, 1 = female)
MOTHED	\makecell[l]{Mother's Education (0 = less than high school, 1 = high school diploma, \\2 = some college,

---

Descriptive Statistics

Input Syntax

Below is input syntax to call descriptive statistics.

```
TITLE: LSAL Descriptive Statistics;

DATA:
  FILE = "LSAL_data.dat";

VARIABLE:
  NAMES = CASENUM COHORT SCHOOLID GENDER RACETH MOTHED ISCIIRT KA47A KA47H KA47I KA47K
   KA47L KA9A KA9B KA9C KA9D KA9E KA9F KA9G KA9I KA9J KA9K KB9H FEMALE URM; !Column Names in order
  MISSING=.; ! Identify missing value
  USEVAR = URM FEMALE MOTHED ISCIIRT KA9B KA9D KA9G KA9K
  KA47A KA47H KA47I KA47K KA47L; ! Select variables to examine
  CATEGORICAL = KA47A KA47H KA47I KA47K KA47L URM FEMALE MOTHED; ! Identify the
  !categorical variables

ANALYSIS:
  TYPE=basic; ! Identified for basic analysis (descriptive statistics)

OUTPUT:
  sampstat; ! Provides descriptive statistics
```

Annotated Output

Sample Statistics

The last part of the output (`UNIVARIATE SAMPLE STATISTICS`), is what we can evaluate and use in our descriptive statistics table for the continuous variables (Table 2).

```
UNIVARIATE SAMPLE STATISTICS


     UNIVARIATE HIGHER-ORDER MOMENT DESCRIPTIVE STATISTICS

         Variable/         Mean/     Skewness/   Minimum/ % with                Percentiles
        Sample Size      Variance    Kurtosis    Maximum  Min/Max      20%/60%    40%/80%    Median

     ISCIIRT              64.101      -0.342      24.440    0.03%      54.610     62.240     64.755
            3592.000     125.578      -0.169      93.130    0.03%      67.390     73.790
     KA9B                  1.855       0.186       1.000   31.32%       1.000      2.000      2.000
            3487.000       0.461      -0.847       3.000   16.86%       2.000      2.000
     KA9D                  1.963       0.050       1.000   25.99%       1.000      2.000      2.000
            3470.000       0.481      -0.923       3.000   22.28%       2.000      3.000
     KA9G                  2.026      -0.033       1.000   22.09%       1.000      2.000      2.000
            3476.000       0.468      -0.862       3.000   24.74%       2.000      3.000
     KA9K                  1.767       0.298       1.000   36.33%       1.000      2.000      2.000
            3476.000       0.440      -0.782       3.000   13.06%       2.000      2.000
```

Proportion and Counts

Earlier in the output (`UNIVARIATE PROPORTIONS AND COUNTS FOR CATEGORICAL VARIABLES`), is what we can evaluate and use in our descriptive statistics table for the categorical variables (Table 2).

```
UNIVARIATE PROPORTIONS AND COUNTS FOR CATEGORICAL VARIABLES

    URM
      Category 1    0.777         4313.000
```

```
  Category 2    0.223         1241.000
FEMALE
  Category 1    0.509         3026.000
  Category 2    0.491         2919.000
MOTHED
  Category 1    0.147          854.000
  Category 2    0.580         3362.000
  Category 3    0.103          597.000
  Category 4    0.118          684.000
  Category 5    0.052          300.000
KA47A
  Category 1    0.466         1564.000
  Category 2    0.534         1793.000
KA47H
  Category 1    0.550         1837.000
  Category 2    0.450         1502.000
KA47I
  Category 1    0.452         1507.000
  Category 2    0.548         1825.000
KA47K
  Category 1    0.659         2200.000
  Category 2    0.341         1139.000
KA47L
  Category 1    0.597         2003.000
  Category 2    0.403         1352.000
```

Note. The sample sizes presented in Table 2 are taken from later outputs to account for missingness in the analyses.

Moderation using the ML Three-Step Method

---

1. Class Enumeration

In the first step of the ML three-step, we decide how many classes should represent the heterogeneity in the set of indicators. First, we start with identifying a one-class model and increasing the number of classes until a nominal increase in model fit or non-identification of the estimated model solution is found. See Nylund-Gibson & Choi (2018) for a comprehensive review on enumeration methods. Below is the syntax for the four-class model. Change the estimation of class by replacing the 4 in `CLASSES = c(4);` and rerun the model in M*plus*.

An important note: Under `SAVEDATA:`, the classification probabilities and modal class assignment are requested to be saved into a new dataset. This is not necessary to enter into the syntax until after the latent class model is selected.

Input Syntax

```
TITLE: LSAL 4-Class Model;
DATA:
  FILE = "LSAL_data.dat";
```

```
  VARIABLE:
    NAMES = CASENUM COHORT SCHOOLID GENDER RACETH MOTHED ISCIIRT
  KA47A KA47H KA47I KA47K KA47L KA9A KA9B KA9C KA9D KA9E KA9F
  KA9G KA9I KA9J KA9K KB9H FEMALE URM;
    MISSING=.;
    USEVAR = AKA47A KA47H KA47I KA47K KA47L;
    CATEGORICAL = KA47A KA47H KA47I KA47K KA47L; ! Identified as categorical for binary LCA
    CLASSES = c(4); ! Class 4
    AUXILIARY = URM FEMALE MOTHED ISCIIRT KA9B KA9D KA9G KA9K; ! Identifying auxiliary variables
  ANALYSIS:
    ESTIMATOR = mlr;
    TYPE = mixture;
    !OPTSEED = 573096; ! set seed to replicate analyses at the same log-likelihood and initial starts
  !SAVEDATA: ! Only keep this when rerunning the chosen latent class model
    !FILE = savedata.dat;
    !SAVE = cprob;

  PLOT:
    TYPE = plot3;
    SERIES = KA47A KA47H KA47I KA47K KA47L(*);
```

Annotated Output

Sample Size

At the beginning of an LCA output, we can see our sample size and the number of dependent and categorical variables used. We are estimating one categorical variable (latent class variable) and five indicator variables.

```
SUMMARY OF ANALYSIS

Number of groups                                                    1
Number of observations                                           3364

Number of dependent variables                                       5
Number of independent variables                                     0
Number of continuous latent variables                               0
Number of categorical latent variables                              1
```

Proportion and Counts

Here, we can see the proportions and counts for each indicator variable, `Category 1` is no endorsement and, `Category 2` is the endorsement of the indicator variables.

```
UNIVARIATE PROPORTIONS AND COUNTS FOR CATEGORICAL VARIABLES

    KA47A
      Category 1    0.466         1564.000
      Category 2    0.534         1793.000
    KA47H
      Category 1    0.550         1837.000
      Category 2    0.450         1502.000
    KA47I
      Category 1    0.452         1507.000
      Category 2    0.548         1825.000
```

```
    KA47K
      Category 1    0.659         2200.000
      Category 2    0.341         1139.000
    KA47L
      Category 1    0.597         2003.000
      Category 2    0.403         1352.000
```

Class Size

Here, we can find class sizes. For example, 10.588% of the sample are in Class 1. *Important note*: each time the model is re-run, there is a chance of the classes rearranging. Always check the class sizes and probabilities (shown next) when referring to the classes. Use `OPTSEED` (See Mplus manual) in the input syntax to set the seed for analysis and avoid class rearrangement.

```
FINAL CLASS COUNTS AND PROPORTIONS FOR THE LATENT CLASSES
BASED ON THE ESTIMATED MODEL

    Latent
   Classes

       1       1007.47449          0.29949
       2        879.57820          0.26147
       3        253.01335          0.07521
       4       1223.93396          0.36383
```

Labels of Latent Class Based on M*plus* Output

**Latent Class**	**Label**
1	Pro-Science with Elevated Utility Value
2	Ambivalent with Minimal Utility Value
3	Ambivalent with Elevated Utility Value
4	Anti-Science with Minimal Utility Value

Conditional Item Probabilities

Below is the output that identifies the conditional item probabilities. The values under `Estimate` are the conditional item probabilities for each indicator variable across each latent class. Recall that `Category 1` is no endorsement and `Category 2` is the endorsement of the indicator variables. For example, the probability of those in *Class 1* endorsing item *KA47A* is 0.593. The endorsement of the conditional item probabilities should be plotted to visualize the latent class variable.

```
RESULTS IN PROBABILITY SCALE

                                                        Two-Tailed
                    Estimate       S.E.  Est./S.E.    P-Value

Latent Class 1

 KA47A
    Category 1         0.107      0.012      9.170      0.000
    Category 2         0.893      0.012     76.391      0.000
 KA47H
```

Category 1	0.061	0.023	2.618	0.009
Category 2	0.939	0.023	40.440	0.000
KA47I				
Category 1	0.015	0.013	1.149	0.251
Category 2	0.985	0.013	74.486	0.000
KA47K				
Category 1	0.169	0.020	8.679	0.000
Category 2	0.831	0.020	42.600	0.000
KA47L				
Category 1	0.040	0.015	2.607	0.009
Category 2	0.960	0.015	63.155	0.000

Latent Class 2

KA47A				
Category 1	0.418	0.033	12.646	0.000
Category 2	0.582	0.033	17.575	0.000
KA47H				
Category 1	0.468	0.038	12.235	0.000
Category 2	0.532	0.038	13.890	0.000
KA47I				
Category 1	0.232	0.036	6.495	0.000
Category 2	0.768	0.036	21.546	0.000
KA47K				
Category 1	0.831	0.024	34.737	0.000
Category 2	0.169	0.024	7.080	0.000
KA47L				
Category 1	0.847	0.080	10.532	0.000
Category 2	0.153	0.080	1.905	0.057

Latent Class 3

KA47A				
Category 1	0.323	0.045	7.219	0.000
Category 2	0.677	0.045	15.104	0.000
KA47H				
Category 1	0.708	0.073	9.713	0.000
Category 2	0.292	0.073	3.998	0.000
KA47I				
Category 1	0.724	0.194	3.737	0.000
Category 2	0.276	0.194	1.425	0.154
KA47K				
Category 1	0.510	0.058	8.859	0.000
Category 2	0.490	0.058	8.517	0.000
KA47L				
Category 1	0.000	0.000	0.000	1.000
Category 2	1.000	0.000	0.000	1.000

Latent Class 4

KA47A				
Category 1	0.825	0.015	56.366	0.000
Category 2	0.175	0.015	11.997	0.000
KA47H				

```
   Category 1         0.980      0.013     77.136      0.000
   Category 2         0.020      0.013      1.546      0.122
KA47I
   Category 1         0.915      0.023     40.150      0.000
   Category 2         0.085      0.023      3.710      0.000
KA47K
   Category 1         0.970      0.006    154.358      0.000
   Category 2         0.030      0.006      4.740      0.000
KA47L
   Category 1         1.000      0.000      0.000      1.000
   Category 2         0.000      0.000      0.000      1.000
```

---

2. Determine Measurement Error

After the enumeration step, the logits for the classification probabilities of the modal class assignment are extracted from the output created in the enumeration step. These logits are used in the third and final step to determine the measurement error of the modal class assignment. There are no models estimated in this step, only the extraction of the logits to be used in the final step.

Annotated Output

Logits for Classification Probabilities

Below is appended output from the enumeration step.

```
CLASSIFICATION QUALITY

Logits for the Classification Probabilities for the Most Likely Latent Class Membership (Column)
by Latent Class (Row)

             1         2         3         4

    1      8.959     6.319     5.092     0.000
    2     -0.610     2.237    -0.819     0.000
    3      4.497     4.353     6.199     0.000
    4     -8.219    -2.150   -13.705     0.000
```

The logits presented are entered manually into the syntax in step three. See the next step on how these logits are included in the syntax.

---

3. Adding Auxiliary Variables

Finally, the new dataset created in the first step (which includes modal class assignment) and the logits extracted in the second step is ready to be used in the third and final step: specifying the moderation model with auxiliary variables. Additionally, we can test the equivalence of the regression intercepts, which in this context is the mean of the distal outcome, using the Wald chi-square test. Moderation occurs when at least one slope is different, as evidenced by a significant Wald chi-square test. However, the omnibus Wald tests must be conducted separately.

Input Syntax

A linear regression of the distal outcome(s) on the predictor(s) is freely estimated across each latent class to test for moderation. In M*plus*, this is done by repeating the regression in each of the class-specific statements. See Figure 1 for the path diagram that corresponds with this syntax.

```
TITLE: LSAL Moderation;
DATA:
  FILE = "savedata.dat";

VARIABLE:
  NAMES = KA47A KA47H KA47I KA47K KA47L FEMALE MOTHED ISCIIRT KA9B KA9D KA9G KA9K URM
     CPROB1 CPROB2 CPROB3 CPROB4 N;
  MISSING=.;
  USEVAR = FEMALE MOTHED ISCIIRT URM KA9B KA9D KA9G KA9K N;
  CLASSES = c(4);
  NOMINAL = N; ! N is the modal class assignment from the dataset we created in step 1

DEFINE:
  ISCIIRT = ISCIIRT/10; ! Scale the predictor
  CENTER ISCIIRT (GRANDMEAN); ! Center the predictor

ANALYSIS:
  ESTIMATOR = mlr;
  TYPE = mixture;
  STARTS = 0;
  ITERATIONS = 1000;

MODEL:
  !Covariates: URM FEMALE MOTHED ISCIIRT
  !Distal: ISSUES
  %OVERALL%
  ISSUES by KA9B KA9D KA9G KA9K; ! Creating the factor for the distal outcome
  ISSUES on URM FEMALE MOTHED; ! Covariates -> Science Issues
  ISSUES on ISCIIRT; ! Science Scores -> Science Issues

         %C#1% ! Class 1
 [N#1@8.959]; ! The modal class assignment variable (N) and logits are entered here
 !to specify measurement error
 [N#2@6.319];
 [N#3@5.092];
      [ISSUES] (B01); ! Estimation of intercept
      ISSUES;
      ISSUES on ISCIIRT(B11); ! Estimation of slope (Science Scores -> Science Issues)


        %C#2% ! Class 2
[N#1@-0.61];
[N#2@2.237];
[N#3@-0.819]
      [ISSUES@0] (B02);
      ISSUES;
      ISSUES on ISCIIRT(B12);


        %C#3% ! Class 3
```

```
  [N#1@4.497];
  [N#2@4.353];
  [N#3@6.199];
        [ISSUES] (B03); ! Here, we set a class equal to zero for measurement identification
        !of the latent factor
        ISSUES;
        ISSUES on ISCIIRT(B13);


          %C#4% ! Class 4
  [N#1@-8.219];
  [N#2@-2.15];
  [N#3@-13.705];
        [ISSUES] (B04);
        ISSUES;
        ISSUES on ISCIIRT(B14);

  MODEL TEST:
      !Omnibus test 1 !Only one omnibus test may be estimate at one time, the second one
! is commented out here. After estimating this first omnibus test of slopes, the second
! omnibus test of intercept may be estimated after removing the "!" the second test
! and commenting out the first test.
       B11=B12;
       B12=B13;
       B13=B14;
      !Omnibus test 2
       !B01=B02;
       !B02=B03;! Because we set class two equal to zero,
! we can not include its intercepts in the omnibus test
       B01=B03;
       B03=B04;

  MODEL CONSTRAINT: ! Pairwise differences for slope and intercepts can be tested simultaneously
      new (slope12, slope13, slope14, slope23, slope24, slope34,
           int12, int14, int24);
      slope12=B11-B12;
      slope13=B11-B13;
      slope14=B11-B14;
      slope23=B12-B13;
      slope24=B12-B14;
      slope34=B13-B14;
      int12=B01-B03; ! Class two not included
      int14=B01-B04;
      int24=B03-B04;
```

Annotated Output

Sample Statistics

Presented are the updated sample statistics accounting for listwise deletion in the analyses.

```
UNIVARIATE SAMPLE STATISTICS
```

UNIVARIATE HIGHER-ORDER MOMENT DESCRIPTIVE STATISTICS

Variable/ Sample Size		Mean/ Variance	Skewness/ Kurtosis	Minimum/ Maximum	% with Min/Max	Percentiles 20%/60%	40%/80%	Median
KA9B		1.875	0.153	1.000	29.68%	1.000	2.000	2.000
	2571.000	0.453	-0.811	3.000	17.19%	2.000	2.000	
KA9D		1.981	0.024	1.000	24.77%	1.000	2.000	2.000
	2560.000	0.476	-0.900	3.000	22.89%	2.000	3.000	
KA9G		2.037	-0.045	1.000	21.22%	1.000	2.000	2.000
	2564.000	0.460	-0.828	3.000	24.92%	2.000	3.000	
KA9K		1.764	0.293	1.000	36.21%	1.000	2.000	2.000
	2568.000	0.433	-0.759	3.000	12.62%	2.000	2.000	
FEMALE		0.518	-0.073	0.000	48.17%	0.000	0.000	1.000
	2591.000	0.250	-1.995	1.000	51.83%	1.000	1.000	
MOTHED		2.402	1.080	1.000	11.04%	2.000	2.000	2.000
	2591.000	1.042	0.466	5.000	5.44%	2.000	3.000	
ISCIIRT		0.000	-0.300	-3.409	0.04%	-0.936	-0.203	0.055
	2591.000	1.195	-0.223	2.827	0.04%	0.317	0.963	
URM		0.196	1.531	0.000	80.39%	0.000	0.000	0.000
	2591.000	0.158	0.344	1.000	19.61%	0.000	0.000	

Slope Differences

Below is the first omnibus Wald test result for slope differences. In this example, this is evidence of a significant moderation because of the significant Wald test. That is, there is a significant relationship between the predictor (science scores) and the distal outcome (interest in science issues) across at least one of the classes, $\chi^2(3) = 11.003, p = .012$.

```
MODEL FIT INFORMATION

Wald Test of Parameter Constraints

          Value                            11.003
          Degrees of Freedom                    3
          P-Value                          0.0119
```

Intercept Differences

Below is the second omnibus Wald test result for intercept differences. There was evidence that there are significant differences in the distal outcome means across the science attitude classes, $\chi^2(2) = 205.616, p < .001$.

```
MODEL FIT INFORMATION

Wald Test of Parameter Constraints

          Value                           205.616
          Degrees of Freedom                    2
          P-Value                          0.0000
```

Pairwise Slope and Intercept Differences

To further investigate which class-specific relations differ, pairwise comparisons of the regression slopes and means of the distal outcome are shown below.

```
MODEL RESULTS

                                                   Two-Tailed
                    Estimate       S.E.  Est./S.E.    P-Value
New/Additional Parameters
    SLOPE12           -0.003      0.028     -0.095      0.924
    SLOPE13            0.032      0.037      0.864      0.388
    SLOPE14            0.072      0.022      3.207      0.001
    SLOPE23            0.035      0.043      0.808      0.419
    SLOPE24            0.075      0.032      2.352      0.019
    SLOPE34            0.040      0.037      1.068      0.285
    INT13              0.187      0.051      3.667      0.000
    INT14              0.383      0.027     14.237      0.000
    INT34              0.196      0.048      4.057      0.000
```

Here, `SLOPE12` is the pairwise difference between the slopes in classes 1 and 2. Class 4 (Anti-Science with Minimal Utility Value) was significantly different from Class 1 (Pro-Science with Elevated Utility Value) and Class 2 (Ambivalent with Minimal Utility Value), $p < .05$. Comparisons across intercepts (or the distal outcome means) are all significant.

Slope and Intercept Coefficients

Additionally, each regression between the predictor and outcome can be examined across classes, as well as the intercept coefficients (*Note*: Recall that the mean of the distal outcome factor, Interest in Science Issues, was set to zero for the Ambivalent w/ Minimal Utility Value for measurement identification when adding the latent variable. This class was used as the reference class, thus the mean of the factor is set to zero, and others are compared to it.)

```
MODEL RESULTS

                                                   Two-Tailed
                    Estimate       S.E.  Est./S.E.    P-Value

Latent Class 1

 ISSUES   BY
    KA9B               1.000      0.000    999.000    999.000
    KA9D               1.247      0.033     37.844      0.000
    KA9G               1.169      0.034     34.802      0.000
    KA9K               0.814      0.029     28.087      0.000

 ISSUES     ON
    FEMALE            -0.160      0.019     -8.513      0.000
    MOTHED             0.004      0.009      0.416      0.677
    URM                0.043      0.024      1.798      0.072
    ISCIIRT            0.149      0.015      9.829      0.000

 Means
    N#1                8.959      0.000    999.000    999.000
    N#2                6.319      0.000    999.000    999.000
    N#3                5.092      0.000    999.000    999.000
```

**Intercepts**				
KA9B	1.896	0.035	54.251	0.000
KA9D	2.009	0.042	47.323	0.000
KA9G	2.061	0.040	51.416	0.000
KA9K	1.782	0.029	60.462	0.000
ISSUES	0.246	0.031	7.952	0.000
**Residual Variances**				
KA9B	0.231	0.009	24.948	0.000
KA9D	0.131	0.008	16.109	0.000
KA9G	0.156	0.008	18.445	0.000
KA9K	0.285	0.010	29.270	0.000
ISSUES	0.145	0.011	12.864	0.000
**Latent Class 2**				
**ISSUES BY**				
KA9B	1.000	0.000	999.000	999.000
KA9D	1.247	0.033	37.844	0.000
KA9G	1.169	0.034	34.802	0.000
KA9K	0.814	0.029	28.087	0.000
**ISSUES ON**				
FEMALE	-0.160	0.019	-8.513	0.000
MOTHED	0.004	0.009	0.416	0.677
URM	0.043	0.024	1.798	0.072
ISCIIRT	0.152	0.023	6.544	0.000
**Means**				
N#1	-0.610	0.000	999.000	999.000
N#2	2.237	0.000	999.000	999.000
N#3	-0.819	0.000	999.000	999.000
**Intercepts**				
KA9B	1.896	0.035	54.251	0.000
KA9D	2.009	0.042	47.323	0.000
KA9G	2.061	0.040	51.416	0.000
KA9K	1.782	0.029	60.462	0.000
ISSUES	0.000	0.000	999.000	999.000
**Residual Variances**				
KA9B	0.231	0.009	24.948	0.000
KA9D	0.131	0.008	16.109	0.000
KA9G	0.156	0.008	18.445	0.000
KA9K	0.285	0.010	29.270	0.000
ISSUES	0.141	0.011	12.633	0.000
**Latent Class 3**				
**ISSUES BY**				
KA9B	1.000	0.000	999.000	999.000
KA9D	1.247	0.033	37.844	0.000
KA9G	1.169	0.034	34.802	0.000

KA9K	0.814	0.029	28.087	0.000
**ISSUES ON**				
FEMALE	-0.160	0.019	-8.513	0.000
MOTHED	0.004	0.009	0.416	0.677
URM	0.043	0.024	1.798	0.072
ISCIIRT	0.117	0.033	3.503	0.000
**Means**				
N#1	4.497	0.000	999.000	999.000
N#2	4.353	0.000	999.000	999.000
N#3	6.199	0.000	999.000	999.000
**Intercepts**				
KA9B	1.896	0.035	54.251	0.000
KA9D	2.009	0.042	47.323	0.000
KA9G	2.061	0.040	51.416	0.000
KA9K	1.782	0.029	60.462	0.000
ISSUES	0.059	0.053	1.105	0.269
**Residual Variances**				
KA9B	0.231	0.009	24.948	0.000
KA9D	0.131	0.008	16.109	0.000
KA9G	0.156	0.008	18.445	0.000
KA9K	0.285	0.010	29.270	0.000
ISSUES	0.175	0.021	8.523	0.000

Latent Class 4

**ISSUES BY**				
KA9B	1.000	0.000	999.000	999.000
KA9D	1.247	0.033	37.844	0.000
KA9G	1.169	0.034	34.802	0.000
KA9K	0.814	0.029	28.087	0.000
**ISSUES ON**				
FEMALE	-0.160	0.019	-8.513	0.000
MOTHED	0.004	0.009	0.416	0.677
URM	0.043	0.024	1.798	0.072
ISCIIRT	0.077	0.018	4.327	0.000
**Means**				
N#1	-8.219	0.000	999.000	999.000
N#2	-2.150	0.000	999.000	999.000
N#3	-13.705	0.000	999.000	999.000
**Intercepts**				
KA9B	1.896	0.035	54.251	0.000
KA9D	2.009	0.042	47.323	0.000
KA9G	2.061	0.040	51.416	0.000
KA9K	1.782	0.029	60.462	0.000
ISSUES	-0.137	0.032	-4.312	0.000

Residual Variances

KA9B	0.231	0.009	24.948	0.000
KA9D	0.131	0.008	16.109	0.000
KA9G	0.156	0.008	18.445	0.000
KA9K	0.285	0.010	29.270	0.000
ISSUES	0.174	0.011	15.175	0.000

End of Annotated Output
